# Sequence analysis of European maize inbred line F2 provides new insights into molecular and chromosomal characteristics of presence/absence variants

**DOI:** 10.1186/s12864-018-4490-7

**Published:** 2018-02-05

**Authors:** Aude Darracq, Clémentine Vitte, Stéphane Nicolas, Jorge Duarte, Jean-Philippe Pichon, Tristan Mary-Huard, Céline Chevalier, Aurélie Bérard, Marie-Christine Le Paslier, Peter Rogowsky, Alain Charcosset, Johann Joets

**Affiliations:** 1 Genetique Quantitative et Evolution – Le Moulon, INRA, Université Paris-Sud, CNRS, AgroParisTech, Université Paris-Saclay, Gif-sur-Yvette, France; 2 BIOGEMMA, Chappes, France; 3 MIA, INRA, AgroParisTech, Université Paris-Saclay, Paris, France; 4 EPGV US 1279, INRA, CEA, IG-CNG, Université Paris-Saclay, Evry, France; 5 Laboratoire Reproduction et Développement des Plantes, Univ Lyon, ENS de Lyon, UCB Lyon 1, CNRS, INRA, Lyon, France

**Keywords:** Maize, European germplasm, Structural variation, De novo assembly, Presence absence variation (PAV), Genetic diversity, Microhomology mediated end joining (MMEJ), Double strand break repair (DSBR), Linkage disequilibrium, Pan-genome

## Abstract

**Background:**

Maize is well known for its exceptional structural diversity, including copy number variants (CNVs) and presence/absence variants (PAVs), and there is growing evidence for the role of structural variation in maize adaptation. While PAVs have been described in this important crop species, they have been only scarcely characterized at the sequence level and the extent of presence/absence variation and relative chromosomal landscape of inbred-specific regions remain to be elucidated.

**Results:**

De novo genome sequencing of the French F2 maize inbred line revealed 10,044 novel genomic regions larger than 1 kb, making up 88 Mb of DNA, that are present in F2 but not in B73 (PAV). This set of maize PAV sequences allowed us to annotate PAV content and to analyze sequence breakpoints. Using PAV genotyping on a collection of 25 temperate lines, we also analyzed Linkage Disequilibrium in PAVs and flanking regions, and PAV frequencies within maize genetic groups.

**Conclusions:**

We highlight the possible role of MMEJ-type double strand break repair in maize PAV formation and discover 395 new genes with transcriptional support. Pattern of linkage disequilibrium within PAVs strikingly differs from this of flanking regions and is in accordance with the intuition that PAVs may recombine less than other genomic regions. We show that most PAVs are ancient, while some are found only in European Flint material, thus pinpointing structural features that may be at the origin of adaptive traits involved in the success of this material. Characterization of such PAVs will provide useful material for further association genetic studies in European and temperate maize.

**Electronic supplementary material:**

The online version of this article (10.1186/s12864-018-4490-7) contains supplementary material, which is available to authorized users.

## Background

With the increasing number of genome sequences, it has become clear that structural variation (SV) between individuals of the same species is more prevalent than previously anticipated [1] and has led to reconsider genomes as more dynamic systems. To better characterize genome dynamics, discovery of SVs among individuals is therefore necessary. SV includes deletions, insertions, duplications as well as translocations and inversions. The term « copy number variation » (CNV) is generally used to define deletions, insertions and duplications larger than 1 kb [2] that are present as at least one copy in any individual. Although the definition of CNVs can be applied to any type of sequence, particular attention has been given to gene CNVs due to their potential phenotypic effect. In human, CNVs are considered as major players in driving human evolution, genetic diversity between individuals, and are held responsible for a large number of traits [3]. They are implicated in genetic disorders [4], but can also have a beneficial phenotypic effect [5]. In plants, much less is known about the association of CNV with phenotype, but examples are emerging. For instance, they are involved in metabolite production, flowering time, submergence tolerance, aluminum tolerance, phosphorus uptake and biotic stress response [6–15]. Presence Absence Variants (PAVs), i.e. sequences that are present in one genome and absent in another, are often considered as extreme forms of CNVs [1]. However, it is not yet known whether PAVs and CNVs share the same genomic features. For instance, a large number of PAVs from various species are involved in biotic stress response [6–8, 10, 12, 14]. There is also evidence that they may originate from particular molecular mechanisms [16]. Hence, PAVs may be original both in their evolutionary dynamics and in their biological impact.

Since the 1940’s, maize has been known to harbor large genetic diversity, in terms of differences in genome size, content and size of heterochromatic knobs, repetitive DNA content and SNPs [17–21]. First insights on SV were given from BAC sequences and revealed extensive presence/absence variation of transposable elements (TEs) in intergenic regions [22]. Some of these were shown to carry genes [23, 24]. Gene CNVs were also identified from other locus-specific analyses targeting specific tandemly repeated gene families, such as the R-complex involved in pigment biosynthesis [25] and the *A1-b* locus [26]. Extension of maize SV discovery at the whole genome scale through Comparative Genomic Hybridization arrays (aCGH)-based analysis of low copy regions detected thousands of PAVs and CNVs between two American maize inbred lines [27, 28]. Probing of structural variation through a global analysis of read-depth in over 100 maize lines showed that over 90% of the maize genome shows some degree of CNV between lines [29]. While they allowed cost-effective discovery of PAVs/CNVs in multiple samples, these aCGH and remapping-based studies did not allow discovering novel regions absent from B73. Comparative transcriptome sequencing analysis from 503 maize inbred lines revealed thousands of transcripts that were not present in B73 but present in other genotypes [30]. This study is limited to expressed gene analysis and did not provide sequence breakpoints. In a comparison of PH207 and B73 transcriptomes, over 2500 genes were found specific to one genotype [31]. Genome sequencing can ultimately provide precise breakpoint positions, distinction between CNV and PAV, access to novel sequences, variant size information and exploration of non-genic space. Targeted assembly of non B73 regions from elite Chinese and American lines led to the discovery of 5.4 Mb of new sequence [32]. However, the low sequencing depth used (5X) limited the reconstruction of full-length PAV sequences, therefore limiting complete annotation and hampering their anchoring to the reference genome, thus impeding breakpoint detection.

Most PAVs described to date in maize are shared among multiple inbred lines and likely predate maize domestication, as suggested by their presence in teosinte, the ancestor of maize [33]. However, the recent finding of a CNV that is rare among maize inbred lines and absent in teosinte shows that CNVs are still arising in the maize genome, at least posterior to domestication but possibly more recently [13]. Interestingly, the three-copy allele of this CNV confers aluminum tolerance and the few maize lines that carry it share the same geographical origin with highly acidic soil, thus suggesting that CNVs may play a significant role in maize adaptation.

The European germplasm derives from the American one, and is known to have experienced a major differentiation from its ancestral material, that was introduced in Europe soon after discovery of America [34]. At least two introductions occurred into Europe in the late 15th and/or early 16th centuries: one from American Northern Flints in the North, and one from Tropicals in the South [34, 35]. These two genetic sources were then hybridized, thus generating European Flints. Considering European maize history, B73 is an incomplete reference for European material [35]. Hence, sequencing genomes of European maize lines is crucial to both uncover the genetic originality of the European material and to generate genome sequences that provide close references for European germplasm analysis.

Following World War II, European landraces were used to develop inbred lines, such as F2 and F7 that were derived from the open pollinated Lacaune Pyrenean population. Such lines were showing an outstanding hybrid vigor (heterosis) when crossed to American lines, thus leading to hybrids that combine both productivity and adaptative features to European climate. This unique “heterotic” pattern still prevails today in Northern Europe material, and deciphering the molecular bases of its success is an important evolutionary question, and a major question for European breeders. French maize inbred line F2 played a key role in European breeding programs over the past 50 years. It has been the parent of hybrid varieties planted on up to 70% of maize growing area in Northern France until the middle 1980s and contributes to the pedigree of numerous lines used now as hybrid parents.

Here, we use massively parallel sequencing to identify patterns of SV between genomes of French maize inbred line F2 and American inbred line B73. Using de novo assembly of F2 Illumina reads, we discovered 88 Mb of novel genomic sequences that are present in F2 but not in B73. PAV sequence annotation revealed 395 putative new coding genes, which transcriptome was analyzed in 12 tissues. Sequence analysis of PAV breakpoints suggests a role of Microhomology-Mediated End Joining-double strand break repair in maize PAV formation. Genotyping of these PAVs in a temperate maize panel reveals that they are mostly ancient, while some are found only in European Flint material and allowed for LD pattern analysis in PAVs.

## Results

### De novo draft assembly of the F2 genome: A powerful tool to identify B73 and F2 non shared regions

To discover new genome-specific regions in European maize, we produced a draft genomic sequence of F2, a French maize line of genome size similar to this of B73 (1C genome size of 2.46 ± 0.01 Mb, see Additional file 1: Table S1). This de novo whole genome assembly (WGA) of the F2 genome was conducted by processing a 90X short-reads dataset with a combination of ABySS and SSPACE (see Materials and Methods). F2 genome size was estimated by K-mer frequency analysis to 2.47 GB, in agreement with flow cytometry estimation. This assembly represents 1597 Mb (i.e., 64.8% of the F2 genome) with a scaffold N50 of 13,895 bp and NG50 of 4289 bp. Quality of this assembly was assessed by searching for 248 ultra-conserved Core Eukaryotic Genes (CEGs) using CEGMA [36]. As a control, the CEGMA search was also performed on the B73 maize reference genome sequence (v2 and v3). Out of these 248 genes, 233 (94%) and 238 (96%) were found in F2 and B73 assemblies respectively, and 81% were found with a complete CDS in F2 (87% in B73 v2 or v3) (Additional file 1: Table S2). We also searched the F2 WGA with a set of 956 universal single-copy plant orthologs using BUSCO (Benchmarking Universal Single-Copy Orthologs [37]), showing again that assembly quality in gene space is very similar to this of B73 AGPv2 and AGPv3 maize genome sequence (Additional file 1: Table S2). Altogether, these assessments highlight the quality of our F2 WGA in genes. Because F2 WGA covers 64.8% of the F2 genome and genes typically represent less than 5% of a maize genome, our F2 WGA clearly extends beyond the gene space. Homology searches using TE databases indeed revealed that 60% of our F2 WGA corresponds to TE sequences (56% for contigs and scaffolds > 1 kb). To further assess the quality of the assembly in gene-rich and TE-rich regions we aligned F2 scaffolds onto three F2 BAC sequences assembled from Roche 454 or PacBio SMRT sequencing (C. Vitte, personal communication). One of these BACs corresponds to the *Bronze* region while the two others are totally depleted of gene and contain mainly nested TEs which are known to be among the most challenging regions of the genome to assemble. Coverage of BACs by F2 WGA scaffolds range from 44 to 80% (Additional files 2, 3 and 4: Figures S1, S2 & S3) and gene space is fully covered. With no exception, contigs are correctly ordered and oriented within scaffolds, and no evidence of chimeric scaffolds or contigs was detected, thus highlighting the quality of the scaffolding and showing that the F2 scaffolds cover large TE-rich regions.

### High abundance of PAVs between B73 and F2

To detect specific genomic regions of each two lines, we used paired-end (PE) reads to search for mapping footprints pointing to deletions. This was done by (i) detection of incongruent insert size (Breakdancer) and (ii) detection of breakpoints (split-read mapping, Pindel). Only variations with size over 1 kb were considered, following the classical threshold used in SV literature. For commodity, sequences found only in one of the two compared genotypes will be designated hereafter as genotype-specific (B73 or F2-specific). This refers to their discovery, but does not prefigure their actual specificity to these genotypes, as they may be shared with other genotypes.

First, we detected F2-specifc regions by aligning B73 and F2 PE reads on the F2 WGA. In this analysis, B73 reads are used to detect deletions in B73 as compared to F2, and F2 reads are used to both (i) filter out false positives SVs (regions detected with both B73 and reads F2 reads) and (ii) ensure SV sequences do not suffer assembly errors (verification that these regions are mostly covered by properly-mapped F2 PE and MP reads, see methods and Additional file 5: Figure S4). Because our aim was to focus on PAVs, we first ensured that the B73 deleted sequences were not present elsewhere in the B73 genome. For this, we filtered out B73 deletions that were at least partially covered by B73 reads, keeping only B73 deletions with B73 read depth of coverage below 5X over at least 70% of the SV size (from mapping of a 40X B73 sequencing data set).

Using this procedure, we obtained 10,044 F2-specific PAVs, which represent 87.4 Mb (Table 1). Among these, 1028 could be unambiguously anchored to the B73 genome, and 1960 were ambiguously anchored (presence of multiple possible positions on the B73 sequence). In addition to these F2-specific regions first identified using SV detection, 7056 F2 WGA scaffolds were not or poorly covered by B73 reads (totaling less than 20% of bases covered) but were too short to include flanking regions shared with B73 genome sequence. They are therefore classified as “incomplete PAVs”. These could not be anchored due to their incompleteness. Size analysis of these three F2-specific region categories revealed that unambiguously and ambiguously anchored PAVs have similar size distribution (median size of 2.4 and 2.1 kb, respectively (Table 1), while “incomplete PAVs” are much larger (median size of 7.6 kb).

**Table 1:** Size distribution of F2 and B73-specific sequences

	F2-specific sequences				B73-specific sequences
	Unambiguously anchored	Ambiguously anchored	Incomplete	Total	Unambiguously anchored
Total number	1028	1960	7056	10,044	691
Cumulative length (Mb)	3.8	7.6	75.9	87.4	3.0
Maximum (kb)	39.9	69.5	86.5	–	42.4
Average (kb)	3.8	3.9	10.8	–	3.6
Median (kb)	2.4	2.1	7.6	–	2.4

To build a synthetic B73/F2 pan-genome sequence we added these F2-specific sequences to the B73 refGen v2 sequence. F2-specific sequences were grouped in two pseudomolecule-like sequences. The first one contains all anchored F2-specifc sequences separated by segments of 100 N and the second one was contains all unanchored F2-specific sequences separated by stretches of 100 N. This pan-genomic sequence was used as a reference for subsequent mapping of short-reads for PAV genotyping.

We also searched for B73-specific regions by aligning F2 and B73 PE reads on the B73 genome sequence v.2 following the same rationale as this presented above (i.e., following the same procedure and criteria but using the B73 and F2 reads sets reciprocally).

This approach evidenced 691 B73-specific regions (Table 1) ranging in size from 1 kb to 42.4 kb (median size 2.4 kb) that make up a total of 3.0 Mb. This number may seem much less compared to the total number of F2-specific regions found (10,044). However, this is mainly due to the fact that a large fraction of the F2-specific regions (70%, accounting for 86.84% of F2-specific regions cumulative size) is not derived from SV detection but rather corresponds to “incomplete PAVs”. Because the B73 genome sequence is delivered as pseudomolecules, we could not detect B73-specific regions that could be classified as “incomplete PAVs” as we did with F2 scaffolds. Therefore, all B73-specific regions should be evidenced by SV detection. Comparison of B73- and F2-specific regions class by class reveals that B73-specific regions have a median size similar to this of unambiguously and ambiguously anchored F2-specific regions (2.4 kb vs. 2.4 kb and 2.1 kb, respectively), while “incomplete PAVs” are much larger (median of 7.6 kb). This suggests the “incomplete PAVs” class found using F2 genome as a source of specific sequence is not well represented in B73, therefore pointing to a lack of detection of large variants using the SV detection method, even when applied on a complete genome sequence. Indeed, we have manually inspected 20 of the putative F2 deletions > 200 kb and all were misclassified genome duplications.

That being said, PAVs derived from the very same SV detection method and criteria share similar metrics (Table 1 and Additional file 6: Figure S5), but are four times more abundant in F2 (1028 and 1960 totaling 2988, with a cumulative size of 11. Mb) than in B73 (691, cumulative size of 3 Mb).

### F2-specific regions show traces of microhomology

**Fig. 1:**
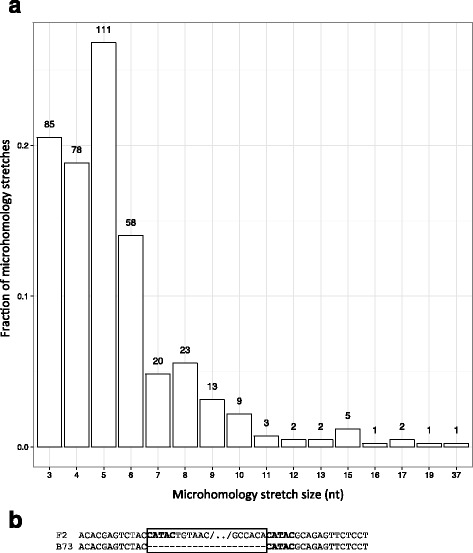
Size distribution of microhomology stretches. **a** Distribution of size of microhomology stretches in PAVs (F2-specific sequences); **b**. Example of a 5 nt microhomology signature in a F2-specific region. Microhomology stretches are shown in bold, and PAV is highlighted by a box

To get insights into the possible molecular origin of genotype-specific sequences, we performed a sequence analysis of breakpoints for the 1028 anchored F2-specific sequences for which a breakpoint was evidenced. Among these, 414 (40%) showed traces of exact microhomology. Because such traces can derive from insertions (with duplication of the target sequence) or deletions (with loss of one site during repair), this analysis by itself did not allow establishing whether F2-specific sequences were F2 insertions or B73 deletions. To address this question, we analyzed the size of the microhomology stretches. As shown in Fig. 1, size of the microhomology stretches ranges from 3 to 37 nt with an average of 5 nt. All but one are less than 25 bp long, a typical feature of Microhomology-Mediated End Joining (MMEJ) mechanisms for Double-Strand Break Repair (DSBR) [38]. Size distribution is globally exponential (Fig. 1), with maximum values for short stretches of microhomologies, a feature expected as short identical stretches of DNA are more frequent than large ones in a genome. Interestingly, however, size distribution of microhomology stretches also shows a second mode with a peak value of 5 bp, which represents 27% of all sequences (Fig. 1). This excess (about 10%) is not expected from DSBR, suggesting that part of the F2-specific sequences originate from another mechanism.

### Uneven distribution of B73 and F2-specific regions in the maize genome

**Fig. 2:**
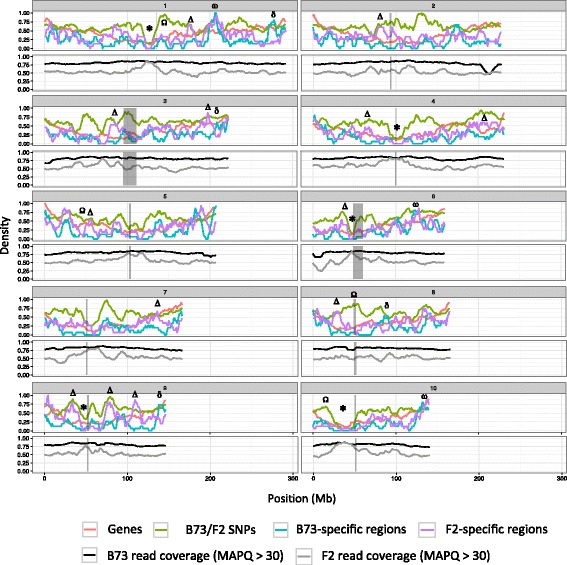
Genomic distribution of B73- and F2-specific regions. PAV distributions along all chromosomes for 10 Mb sliding windows and 1 Mb steps. Each panel represents a different chromosome with chromosome number indicated on top. Grey boxes indicate position of peri-centromeric regions. For each chromosome, top panel: fraction of window covered by B73 genes, number of F2/B73 SNPs per window, PAVs (F2-specific and B73-specific regions). Numbers are scaled relatively to the highest value across the whole genome for each feature type. Bottom panel: fraction of window covered by B73 reads and F2 reads with mapping quality > 30 (no scaling was applied). Asterisks highlight regions of low diversity between F2 and B73. Greek letters represent regions with particular patterns (from visual inspection). Regions with **Ω** abundant SNP and scarce PAV; **ω** abundant SNP and abundant PAV; **Δ** abundant F2-specific regions and scarce B73-specific regions; **δ** scarce F2-specific regions and abundant B73-specific regions

To get insights into their genomic localization, we investigated the distribution of the 1028 unambiguously anchored F2-specific PAVs and 691 B73-specific PAVs along the maize chromosomes (RefGen_v2) using 10 Mb sliding windows (sliding step: 1 Mb). As depicted in Fig. 2, at a broad scale, PAV content follows that of gene along chromosomes. Both F2 and B73-specific regions are not randomly distributed along the genome, with regions of low and high density. In general, PAVs tend to be more abundant at chromosome tips (with the exception of right arm of chromosome 2, chromosome 3 and chromosome 10 left arm). Most pericentromeric regions are globally depleted in F2-specific regions, with the exception of chromosomes 2, 3 and 7. Interestingly, in chromosomes 1, 4, 6, 9 and 10, centromeric regions harbor both a lack of PAVs, a lack of SNPs and a high read mapping quality, thus highlighting regions of high identity between B73 and F2 (Fig. 2, regions marked by asterisks). While PAV global distribution is similar for B73 and F2, local densities of B73 and F2-specific regions are not fully associated, with several regions exhibiting opposite density trends (Fig. 2, regions marked by Δ and δ). This pattern is not attributable to local differences in mapping quality between the two genotypes. At the chromosomal scale, PAV and SNP densities are globally associated (Fig. 2). Several regions exhibit very high SNP and PAV density (marked by ω in Fig. 2), denoting region highly variable between B73 and F2. We also found regions with high SNP density but low PAV density (marked by Ω in Fig. 2). This pattern is especially marked for a large region of chromosome 10 and does not seem to be attributable to major bias in read mapping.

### F2-specific regions contain genes and transposable elements

To get a first insight onto the genomic content of F2 novel sequences, we first estimated their gene and TE content. F2 mRNAseq data generated from 12 tissues (Additional file 1: Table S3) as well as leaf mRNAseq data from other European maize genotypes was used to predict genes in the F2 novel sequences. Predicted genes with a FPKM value over 1 were considered as expressed, leading to identification of 2413 transcribed regions (mean size: 1.73 kb) from the 10,044 F2-specific sequences. Removal of TE-related sequences and selection of genes with peptide prediction length > 100 amino acids led us to identify 417 putative novel genes.

For B73, sequence-specific annotation was based on overlap with existing annotation. B73 regions missing in F2 overlap 238 genes from the maize filtered gene set, among which 91 (38%) lack at least 50% of CDS and 60 (25%) are fully deleted (Additional file 1: Table S4).

Homology-based analysis of TE content revealed that 69% of the F2-specific contigs are made of TEs. With the same methodology, 79% of the B73 genome (V2) and 56% of the F2 WGA (contigs and scaffolds > 1 kb) correspond to TE sequences.

### F2-specific regions contain non-ubiquitous genes

To further analyze the set of new genes found in the F2-specific regions, we used our set of 417 new putative genes to analyze their coding properties and expression patterns. First, we expected that some large PAVs overall specific to one line might contain short coding sequences shared between B73 and F2. So we filtered out PAV predicted coding sequences exhibiting > 99% identity with B73 mRNA over their full length. Twenty-two of such genes were filtered out, thus leading to a final set of 395 novel coding sequences (average size: 1.1 kb). A putative function was then assigned to 91 of these proteins using protein domain annotation and similarity search against UniProtKB/Swiss-Prot sequence database (Additional file 1: Table S4). Annotation of protein functions revealed that 17 of them (20%) are putatively involved in stress response and plant defense, 11 (12%) in biosynthetic processes, 10 (12%) in development, 5 (6%) in protein synthesis and 5 (6%) in chromatin remodeling. For B73, PAV annotation was based on existing RefGen V2 5a annotation, which provided a molecular function for 25 B73-specific genes. Grouping of these molecular function highlighted 6 sequences (25%) putatively involved in metabolism, 4 (16%) in stress response and plant defense, 4 (16%) in protein degradation and 2 (8%) in cytoskeleton/microtubule (Additional file 1: Table S5).

**Fig. 3:**
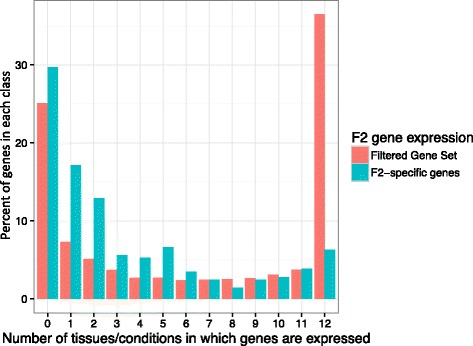
Expression profile F2-specific genes. The number of F2-specific genes (green) expressed in none to all (12) sampled tissues is compared to the number of F2 genes shared with B73 (filtered gene set, red). As gene prediction in F2-specific regions was performed with mRNAseq support from F2 and several additional genotypes, some F2-specific genes can be found not expressed in F2 genotype for the 12 condition tested

Expression of F2-specific genes was analyzed by mapping F2 mRNAseq short-reads onto the B73/F2 pan-genome sequence. F2-specifc genes breadth of expression was compared to this of F2 genes shared with B73 (FGS) (Fig. 3). More than 35% of F2 genes shared with B73 were found expressed in all tissues, while this was true for only 6% of the F2-specific genes. For each tested tissue, F2-specific genes showed a lower average gene expression compared to F2 genes shared with B73 (FGS) (Additional file 7: Figure S6).

### Conservation of F2-specific genes in maize and other grasses

Finally, we investigated the conservation of the 395 F2 novel genes with other maize inbred lines and closely related species using BLASTN analyses onto the NCBI non-redundant nucleotide database (Additional file 1: Table S6). We focused only on BLAST best hits to determine whether the sequence is present in other maize or in close maize relatives. Of these sequences, 358 (90.6%) have a best hit in maize and 31 (7.8%) in other related grasses (20 in *Sorghum*, 3 in *Setaria*, 3 in *Saccharum*, 1 in *Miscanthus*, 1 in *Panicum*, 1 in *Tripsacum*, and 2 in *Oryza*). Among the 358 genes with a maize homology, only 22 (6.1%) shared > 95% nucleotide identity over > 85% of their sequence with B73, and 31 (8.6%) with other maize lines. The remaining 305 sequences exhibit significant similarity (e-value < 1.00E-5) but the region of homology is restricted to a limited part of the F2-specific gene sequence.

### PAVs genotyping is consistent with known maize genetic groups

**Fig. 4:**
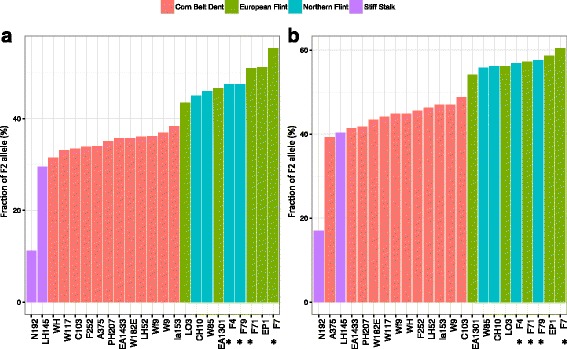
Proportion of F2-present and B73-present PAVs in the core set of 23 maize lines. **a**: Proportion of PAVs, typed as F2 allele (Presence allele is typed). **b**: Proportion of PAVs, present in B73, (Absence allele is typed). Only PAVs with confident genotyping in all lines are represented. Proportions are in percent. Each bar represents one inbred line, with name indicated at the bottom. Colors highlight the 4 genetic groups represented in our core panel of 23 maize inbred lines. Inbred lines are ordered by number of shared variants with F2, from lower (left) to higher (right). B73 (0%) and F2 (100%) are not shown. Asterisks highlight inbred lines of French origin (for details on inbred line origin, see Additional file 1: Table S7)

To investigate the degree of polymorphism of B73/F2 PAVs, we analyzed their presence/absence by mapping reads from 23 temperate maize lines (Additional file 1: Table S7) kindly provided by the CORNFED Plant-KBBE program (PI A. Charcosset) on our B73/F2 pan-genome sequence containing B73 v2 genome sequence plus two additional chromosomes corresponding to anchored and not anchored F2-specific sequences identified in this work (see above). B73 and F2 reads were also included as controls. Read counts were used for scoring presence/absence of B73 and F2-specific regions using a statistical method that we developed (see Material and Methods). About 80% of the PAVs could be classified as present or absent in each inbred line at a BFDR nominal level of 1%. We then investigated what proportion of the F2-specific and B73-specific variants was found in each tested inbred line (Fig. 4).

As expected, a large number (43% to 55%) of the novel sequences discovered in F2 were also present in other European Flints - the group to which F2 belongs - whereas only a small number (11% to 29%) was detected in Stiff Stalks - the group to which B73 belongs (Fig. 4, left). The highest number of shared variants with F2 was found for F7 (55% shared variants), followed by EP1 (51%) and F71 (51%). Northern Flints were found intermixed with European Flints, with French Northern Flints (F79 and F4) sharing more variants with F2 than either a Swiss (CH10) or a Canadian (W85) inbred. Interestingly, the 5 French (asterisks) and Pyrenean (EP1) lines tested shared the most variants with F2, independently of their grouping. Among European and Northern Flints, Italian line LO3 was found to be the most distant to F2. On the other extreme, the lowest number of shared variants with F2 (highest with B73) was found for N192 (11%) (Fig. 4, left). Corn Belt Dents showed a very low variation (10%) in F2 variant proportion, and harbors more PAVs corresponding to B73 alleles (absence) than F2 alleles (presence).

Analysis of the B73-specific variants revealed similar pattern (Fig. 4, right), with only minor differences: LH145 Corn Belt Dent line was found intermixed with Stiff Stalks, and positions of EA1301 and LO3 were interchanged. Interestingly, a larger variability was found among European/Northern Flints using F2-specific regions (12% variation) than using B73-specific regions (7% variation), while the opposite was true for Stiff Stalks (19% vs. 22%). No obvious difference was observed for Corn Belt Dent.

**Fig. 5:**
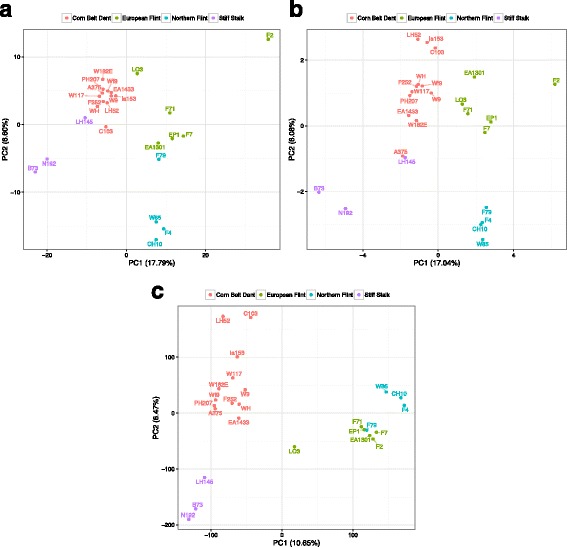
Principal Component Analysis of the 25 inbred lines. Principal Component Analysis based on genotyping of (**a**) F2-present PAVs, (**b**) B73-present PAVs and (**c**) B73/F2 SNPs. Colors highlight the four main maize temperate genetic groups according to [46]

To get further insights into PAV-based genotype proximity, we then used presence/absence data in a PCA to analyze the genetic structure of the 25 inbred lines. We compared the patterns observed using PAVs originally found present in F2 or present in B73 as well as with B73/F2 SNPs found in the shared regions. As shown in Fig. 5, all three analyses separated the 25 lines into the four groups to which they belong, with slight overlap between Northern Flints and European Flints when SNPs and F2-specific regions are considered. No major difference was found between PAVs and SNPs clustering.

### Most PAVs are shared among maize groups but some are found only in European germplasm

**Fig. 6:**
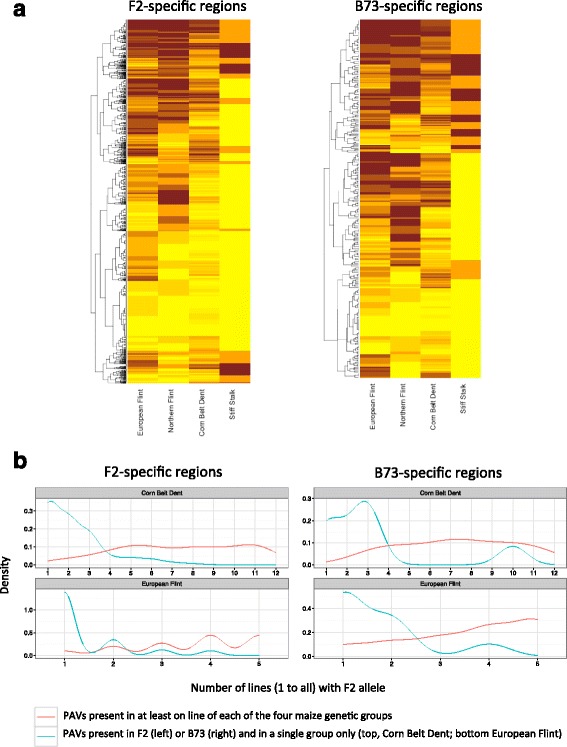
PAVs frequencies in maize genetic groups. **a** Hierarchical clustering of PAV frequency (F2 allele) within maize groups. Left: F2-present PAVs (typing of Presence variant). Right: B73-present PAVs (typing of Absence variant is shown). Horizontal lines represent PAVs. Vertical bars represent the four maize genetic groups. Light colors highlight low frequencies and strong colors indicate high frequencies of the F2 allele. **b** F2-present PAV frequency (left) or B73-present PAV frequency (right) within 2 genetic groups: Corn Belt Dent and European Flint. PAVs shared by F2 and a single genetic group (green) are separated from PAVs shared in at least one individual of the 4 genetic groups analyzed in this study (red). Left: sequences present in F2 and absent in B73. Right: sequences present in B73 and absent in F2

Hierarchical clustering performed on 4218 PAVs present in F2 and absent in B73 with no missing data (Fig. 6a) revealed a majority of cases with high frequency in European Flints and lower frequencies in other groups. Among these, 396 variants (9.3%) are found only in European Flints among which 134 (3.2%) variants are found only in F2 and no other inbred line. On the other extreme, 1064 (25%) of the PAVs present in F2 and absent in B73 are shared among all groups and may correspond to ancient variants with recent losses more or less specific to B73. Another 241 variants (5.7%) are found only in European Flints and Northern Flints, with some found at higher frequency in Northern Flints than in European Flints. Similar analysis using B73-present PAVs also highlighted PAVs common to all groups and PAVs specific to Stiff Stalks (Fig. 6a). More detailed analysis of B73-present PAVs was however restrained by the low number of Stiff Stalk lines analyzed in this study.

To get further insights into PAV frequencies, we classified them into two classes: those present in at least one inbred line of each group, and those present in F2 plus another line of a single group (for PAVs present in F2) or B73 plus another line of a single group (for PAVs present in B73). As shown in Fig. 6b, the first class is depleted in low frequency PAVs while the second is enriched in low frequency PAVs, showing that PAVs specific of a group are also mostly found at low frequency in this group. Interestingly, 46 PAVs were found present in over half of the European Flints lines and in no other group. Another 134 PAVs were found present in F2 only and may represent recent PAVs, or may trace the contribution of ancestors specific to F2.

### PAVs exhibit strong internal LD

**Fig. 7:**
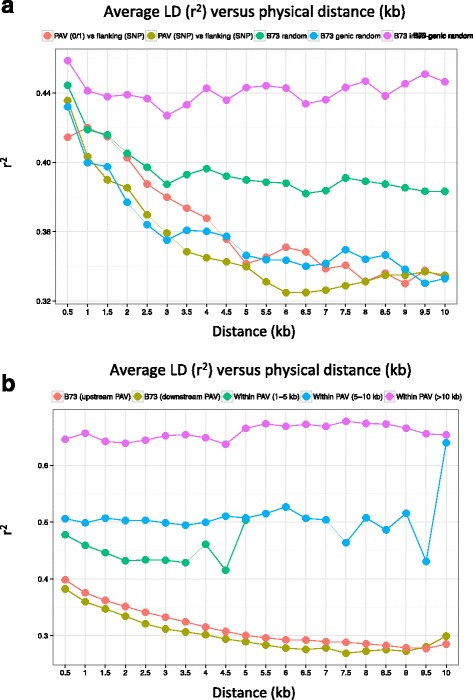
Average LD decay within PAV and between PAVs and their flanking regions. **a** Average LD between PAVs and their flanking genomic regions. Flanking regions is genotyped using SNPs, PAV is genotyped either as 0/1 (red) or using the within-PAV SNP closest to the breakpoint (kaki). For comparison, LD in random maize regions (green), maize random regions from gene space (blue), and maize random regions from inter-genic space (purple) are also plotted. **b** Comparison of average LD within PAVs to LD within flanking regions (upstream, red and downstream, kaki). PAVs are separated in 3 classes of size, 1–4 kb (green), 5–10 kb (blue), 10–40 kb (purple). LD is estimated by the squared correlations of allele frequency (*r*^2^) and plotted against distance between polymorphic sites (0 to 10 kb)

For the 1028 novel F2 sequences that were unambiguously anchored to the B73 genome sequence, we investigated LD decay between F2-specific sequence and their flanking regions. For this, LD was measured between the PAV (with presence/absence being estimated as 0/1 or by polymorphism of one internal SNP) and several distant SNPs of the flanking regions (see Materials and Methods). Whatever the PAV typing method (0/1 or SNP-based), LD decay resembles this of random genes of B73 (Fig. 7a) thus suggesting the PAVs analyzed are mostly located in gene space. On average, r^2^ is only about 0.37 for a distance of 2.5 kb, thus highlighting that only closely linked SNPs can capture PAV presence/absence.

Then, to investigate whether genome-specific sequences have a particular LD pattern, we compared LD patterns within PAVs (using internal SNPs) and within their flanking regions (upstream and downstream). As shown in Fig. 7b, LD is strong within F2-specific regions (r^2^ between 0.4 and 0.7) and does not decrease much whatever the size of the PAV. In contrast, flanking regions show a slight decrease with a r^2^ of about 0.35 for a distance of 2.5 kb. It is worth noting that although PAVs are mainly located in gene rich regions, this strong difference of LD patterns within PAVs and within flanking regions should not be affected by genomic location, as the comparison is made at a local scale.

## Discussion

### Discovering new maize genomic regions

Several methods have been described for plant pan-genome sequence assembly, such as direct comparison of genome de novo assemblies, simultaneous assemblies of several genomes into colored de Bruijn graph, and iterative assembly (see [39] for description of methods). Here, we identified B73 and F2-specific regions by a combination of de novo assembly of the F2 genome and structural variation discovery from read mapping.

All these methods rely at least partly on whole genome assemblies and are sensitive to their quality (gaps, repeat and gene family collapsing or expansion, chimeric regions [40]). Besides, read mapping-based SV detection is affected by quality assembly as well as by mapping bias in repeated region of the genome (Paralog confusion, poor mapping in repeated regions) [39, 41].

The F2 whole genome assembly totaled 1.6 Mbp with a scaffold N50 of 13.9 kb. This assembly is of good quality in the gene space. Besides, it covers about 65% of the estimated F2 genome size, clearly showing that we managed to assemble the F2 genome sequence beyond the gene space. Therefore, this genome sequence can be used to detect PAVs in both genic and non-genic regions. Nevertheless, due to the fragmentation and incompleteness of the F2 genome assembly, we took an important care to provide high quality variants, by applying several filters to avoid false positives.

In particular, (i) SV detection was based on reads with good mapping quality, (ii) we ensured that SVs were located in well assembled regions by discarding cases where SV was also detected in self-mapping experiments and by verifying that detected regions had good coverage of properly-mapped reads in self-mapping experiment, as proposed in [40], (iii) we confirmed genome-specificity of PAVs by verifying that the corresponding sequences is no or poorly covered by reads from the genotype in which the region is absent. This stringent procedure was used to produce a set of robust PAVs at the cost of sensitivity.

We have identified over 87 Mb of novel F2 sequences, which is roughly ten times and 12 times more than observed in rice [15] and Arabidopsis [42] respectively, reflecting genome size variation between these species. These novel sequences correspond to 10,044 genome regions, including 395 novel genes. This is close to what was previously observed in maize [32], Arabidopsis [42] and rice [15] (see [39] or further comparison of pan-genome in plant). When comparing PAVs detected with the same method in both genotypes, we evidenced 4 times less B73-specific regions than F2-specific regions. As the F2 assembly is less complete than the B73 one we would have expected the opposite. This suggests that specific genomic regions are more abundant in F2 than in B73. A similar trend emerged from a comparison of B73- and PH207-specific sequences (over 1.3 time more specific genes in PH207 than in B73 [31]).

### Some F2-specific genes may be involved in stress response and plant defense

In previous definition of pan-genome, PAVs have been defined as part of the “dispensable” genome because they are not necessary for survival [43]. As acknowledged later by the authors, this does not mean that PAVs do not contribute to plant phenotypic diversity, and actually may be involved in the interplay between genome and environment, therefore pointing to a possible role in adaptation [44]. As shown by diverse studies [6–15], PAVs and CNVs are often enriched in genes involved in biotic and abiotic constraints. But only one clear example of CNV has been linked to plant adaptation [13]. Consequently, a major question in plant evolution is to decipher to which extent CNVs and PAVs do indeed contribute to plant adaptation. Here, we show that PAV genes are putatively involved in stress response and plant defense, in biosynthetic processes, in development, in protein synthesis and in chromatin remodeling. This, together with the observation that expression of most F2 novel genes is limited to certain conditions/tissues, a trend consistent with this observed for other maize gene PAVs [31], support that at least some of the genes from non-shared genome fraction might be involved in plant adaptation in maize. These genes are good candidates for further analyses.

### F2-specific sequences includes transposable elements

Our F2 genome sequence assembly contains 60% of TE sequences. Because this is less than observed in B73 (85% in [45], 79% using the same methodology as this applied to the F2 genome assembly), we anticipate that our F2 genome assembly is missing some TEs, for instance highly identical and repeated TEs that likely hamper local assembly, or TEs refractory to PCR amplification such as Helitrons (Ed Buckler, personal communication). While it likely does not contain the complete repertoire of TEs, our F2 assembly provides an unprecedented tool to investigate biological features of PAVs without restricting study to gene space only.

By our definition, any PAV made up of repetitive sequences present in B73 was considered as CNV rather than PAV and excluded from our analysis. Therefore, most of TEs in PAV may be divergent old copies and/or may be due to new repetitive sequences present in F2 and absent from B73. Due to our filtering criteria, some may also correspond to low copy regions separated by short stretches of repeats. Considering that these regions could be assembled, we anticipate that the repeated regions they contain are not highly repeated in F2, and/or not highly identical in sequence.

### PAVs underlying mechanisms

Among the 1028 deletions for which a breakpoint was evidenced, 414 (40%) exhibit clear traces of microhomology. While underlying mechanisms still need to be clearly identified, this suggests the possible role of microhomology-mediated end joining (MMEJ) double strand break repair in the origin of these PAVs. This support the idea that a fraction of the F2-specific regions is the result of MMEJ-type DSBR, and therefore corresponds to deletions in B73 rather than to insertions in F2. Such microhomology patterns have also been observed for CNVs in barley [46] and cucumber [47], thus reinforcing the idea that non homologous end joining (NHEJ) is an important factor for genome shrinkage and influences the evolution of plant genomes [48]. Similarly, studies on maize genome fractionation after whole genome duplication suggested short direct repeats to be involved in gene deletions from duplicated regions [16]. However, the signatures observed cannot rule out the implication of insertion rather than deletion processes. While microhomology stretches involved in double strand break repair can be of various sizes [49], we observed an enrichment of 5 bp traces. Because LTR retrotransposons are known to induce 5 bp duplications at insertion [50], we believe that part of the F2 novel regions could correspond to insertions of novel LTR retrotransposons with little or no similarity to those contained in B73. This is in accordance with the observation of high amounts of TEs in F2-specific sequences.

Discovery of 395 new genes allowed us to investigate their conservation within maize and other related species and therefore helped us characterize their molecular origin. For most of new genes, a relative (as complete gene or gene domain) was found either in maize or in a related grass species, confirming they correspond to real genes. However, only some gene exhibited high identity (> 95% identity over 95% of sequence length) with B73 or another maize line, the vast majority (85.2%) showed significant but lower sequence similarity that was often restricted to a subset of the sequence. Therefore, while some PAVs may be members of large gene families as already observed [31, 33] and may contribute to quantitative phenotype variation, others may correspond to unique genes whose deletion may contribute to major qualitative defect. These PAV genes may have originated from differential retention of duplicated gene copies after the last whole genome duplication event in maize evolution [16]. Further analysis of gene completeness will allow to elucidate whether some cases originate from partial gene duplication, possibly mediated by transduction of gene fragments by transposable elements [51] or other mechanisms.

### PAVs chromosomal organization and linkage disequilibrium

Knowledge of evolutionary mechanisms involved in the formation and retention of PAVs remains limited. Here, we could investigate simultaneously the behavior of the genome-specific fractions from two different inbred lines. Of course, one limit of this comparison is the different quality of both B73 and F2 genome assemblies. However, because detection of structural variants depends on mapping quality, it is likely to be inefficient in highly repeated regions whatever the quality of the reference sequence. SV detection will be easier in low or moderately repeated genome regions, where our F2 assembly is of good quality. Therefore, we anticipate F2 and B73 SVs are comparable in the low/middle repeated regions of the genome, and that we should be able to compare global chromosomal patterns. In our study, both B73 and F2-specific regions globally show depletion around pericentromeric regions and an increased density towards chromosome tips, thus globally reflecting gene densities. This pattern is in agreement with what has been observed in a comparison between genomes of B73 and PH207 [31]. This distribution is also in agreement with the observation that LD decay in PAV flanking regions resembles this of genic regions. These chromosomal landscape and LD patterns relate to the subset of PAVs (10%) that could be unambiguously anchored, and may also reflect the fact that anchoring is easier in low-copy genome regions. Whether other types of PAVs show the same pattern remains to be elucidated by directly comparing complete whole genome sequence.

Co-occurrence of high densities of SNPs and PAVs highlights at least three regions of high genetic divergence between B73 and F2 in chromosomes 1, 6 and 10. In contrast, several large regions exhibit low (almost null) genetic variability between B73 and F2 in both SNPs and PAVs, and likely correspond to regions identical by descent between the two lines. Interestingly, all of these are located in or near centromeres, and may be attributable to the low recombination rate in these regions. Besides these few regions, which are more the exception than the rule, most of the genome harbors contrasted density patterns between PAVs and SNPs, as well as between B73 and F2-specific regions. For example, chromosome 10 exhibits an intriguing pattern of high SNP and low PAV except at its right end. Analysis of microhomology stretches suggests at least two different mechanisms leading to PAVs: MMEJ-based deletions and insertions of TEs. This, together with the observation of unambiguously anchored PAV and incomplete large PAVs, suggest that PAVs may occur through several different molecular mechanisms. So generation of PAVs may explain part of the chromosomal landscape observed. Of course, differential selection between SNPs and PAVs and difference of recombination rates between lines may also contribute to the differences observed, as well as different evolutionary history between B73 and F2. Further whole genome comparisons of several maize lines should help decipher the contribution of these various evolutionary forces onto PAVs formation and retention.

For the first time, we could investigate LD patterns in both PAVs and their flanking regions. This analysis revealed that LD is very high and decreases slowly within PAVs, suggesting low recombination rate within these regions. This pattern is in accordance with the intuitive idea that the absence of the corresponding sequence in some inbreds may prevent PAVs to recombine in 100% of the crosses. Interestingly, the contrast between LD pattern within PAVs and within flanking regions suggests that while the absence of these regions in some inbreds impacts recombination within the PAV, it does not affect recombination in surrounding regions.

### Novel F2 regions contribute to the genetic differentiation of European maize

Typing of F2-specific regions in 23 maize lines representing the genetic diversity of temperate maize allowed estimating the degree to which F2 new regions are shared with other American and European lines. F2 novel regions were more shared with other European Flints than with any other inbred and only a small number of them was detected in the Stiff Stalk group, to which belongs B73. Corn Belt Dents were intermediate and showed more variants in common with B73 than F2. Inbred lines from France or close proximity (Pyrenean) share more variants with F2 than lines from any other origin, independently to their classification into European Flint and Northern Flint groups (Fig. 4), thus reflecting the history of the French germplasm.

PAV frequency analysis within genetic groups revealed that most F2-specific and B73-specific sequences are likely of ancient origin. This is in accordance with previous results in maize and teosinte [33]. For instance, a large proportion of PAVs is shared between F2 and at least another European Flint or one Corn Belt Dent line. Because European Flints and Corn Belt Dents are the two groups with higher sampling number, we believe that most of these PAVs also likely correspond to ancient PAVs that are shared between all groups but not detected in the smallest groups due to sampling issues. On the other hand, we also found 396 PAVs present in F2 that are shared by European Flints only and 134 PAVs found present in F2 only. These putative European-specific sequences may correspond to highly divergent regions among maize lines, specific gene retention in F2 following whole genome or gene specific duplication, or transposon burst in F2 with loss of the original copy in the other lines. Further analysis of genomic content, molecular origin and selection pattern of these PAVs are ongoing to discriminate among the possible scenarios. On the other hand, we also found 46 PAVs that are present in more than half of the European Flint lines analyzed and present in no other line, therefore pointing to regions that may have been recently inserted or less frequently deleted in European maize lines compared to American ones. It will be interesting to investigate whether they correspond to regions under selective constraints.

### Towards building a maize pan-genome

Despite the remarkable literature available on maize structural variation [22, 27, 28, 30, 32, 33, 52, 53], and the emerging collection of non B73 maize genome sequences, to our knowledge no maize pan-genome sequence in the form of a draft reference sequence has been constructed and delivered to date. Indeed, how to combine these new sequence resources as a reference for efficient mapping of shotgun reads from large maize collections (for instance for variant calling) is still an open question for the maize community. For instance, mapping on each new genome separately is possible but time consuming and will likely not be effective when many complete genome sequences will be available (Ed Buckler, pers. Comm.). Here, using a methodology retrieving specific parts of each genome sequence, we provide a first draft of a pan-genome sequence for the maize lines B73 and F2. This pan-genome includes the B73 RefGen V2 sequence plus two additional pseudomolecules containing respectively the F2-specific sequences anchored and not anchored to B73 sequence. The methodology can be easily applied to include additional genome sequences or future improved version or B73 and F2 genome assemblies. While neither B73 or F2 genome assembly are complete, the provided B73-F2 pan-genome sequence is an unprecedented material to better characterize and use the European maize germplasm. It should for instance help in completing reference database in view of genotyping by sequencing (GBS) or contribute to the definition of new genotyping arrays to increase the power of genome-wide association studies (GWAS). Considering the history of maize, we expect that sequences from F2 will not be sufficient to well represent the genomic diversity of the European germplasm. Notably, although F2 and F7, (another major ancestor of European maize breeding programs), derive from the same original population (Lacaune), only 55% of PAVs present in F2 are also present in F7. This is in accordance with previous diversity analyses of this population [54] and highlights the need of sequencing other European maize lines. Interestingly, the 5 European Flints and Northern Flints lines used in this study showed a large range of variability in term PAV present in F2 number (8%), thus highlighting the variability present in the European Flint/Northern Flint germplasm. The range of variability was more limited when using B73 present PAVs (4%) revealing the added value of intra-group genome sequences for intra-group characterization. A similar trend is observed for Stiff stalks with PAVs present in B73 (Fig. 4). This suggests that some PAVs present in F2 are present in Corn Belt Dents and absent from Stiff Stalks, thus highlighting that F2 sequencing also provides interesting material for non-European germplasm such as Corn Belt Dents. Considering the high variability existing within the European Flints and Northern Flints analyzed here, we are generating de novo assemblies for several additional European genotypes to provide a large number of non-redundant sequences that will be useful to the maize community in the quest of the complete pan-genome of the species *Zea mays*.

## Conclusions

We built a F2 genome de novo assembly that allows exploring PAVs beyond genic regions, and built a first European/American pan-genome sequence draft. Extraction of PAV sequences allowed us to make a detailed analysis of PAV content and breakpoints, highlighting the possible role of MMEJ-type DSBR in PAV formation and discovering 395 new genes with transcriptional support. Through a genome-wide comparison of two maize inbred lines using de novo assembly, we show that maize genomes have undergone insertions/deletions in different genomic regions. Our results about LD in PAVs support the expectation that PAVs have experienced less recombination than other part of the genome. The finding of genomic regions specific of European Flints also provides another piece of evidence that European material largely differs from its American counterpart.

This in-depth analysis of presence/absence variants between the reference inbred line B73 and the French inbred line F2 enabled us to study the biology of genotype-specific regions and provides unprecedented material for use in European and other maize breeding programs. These resources will for instance help transcriptome and methylome studies of European maize as well as genotyping of the European germplasm to increase the power of GWAS studies. Future association studies using F2-specific sequences will allow deciphering whether these regions have played a role in maize adaptation to European environmental conditions.

## Methods

### F2 genome size estimate

Genome size of the F2 inbred line was estimated by flow cytometry. The total nuclear DNA amount was assessed by flow cytometry according to [55]. *Pisum sativum* L. cv Express long (2C = 8.37 pg) was used as an internal standard. Leaves of the internal standard and maize F2 seedlings were chopped using a razor blade in a plastic Petri dish with 1 ml of Gif nuclei-isolation buffer (45 mM MgCl2, 30 mM sodium citrate, 60 mM MOPS, 1% (*w*/*v*) polyvinylpyrrolidone 10,000, pH 7.2) containing 0.1% (w/v) Triton X–100, supplemented with 5 mM sodium metabisulphite and RNAse (2.5 U/ml). The suspension was filtered through 50 μm nylon mesh. The nuclei were stained with 50 μg/ml propidium iodide, a specific DNA fluorochrome intercalating dye, and kept 5 min at 4 °C. DNA content of 5000–10,000 stained nuclei was determined for each sample using a cytometer (CyFlow SL3, Partec-Sysmex. Excitation 532 nm, 30 mW; emission through a 630/30 nm band-pass filter). The total 2C DNA value was calculated using the linear relationship between the fluorescent signals from stained nuclei of the F2 maize inbred line and the internal standard. The mean value was calculated from measurements of samples comprising at least 3 individuals according to populations.

### DNA sampling and sequencing

Total genomic DNA was extracted from seedlings grown from the F2 INRA seed lot provided by INRA Saint Martin de Hinx. F2 seed lot was validated using 8 microsatellite-derived PCR markers routinely used to discriminate maize inbred lines (D. Madur, personal communication). Extractions were performed using the Macherey-Nagel plant maxi extraction kit using manufacturer’s recommendations. Three types of libraries were produced and sequenced to reach an overall genome coverage of 90X. One hundred one bases paired-end reads (400 bp insert size, 40× genome coverage) were generated by Integragen (Evry, France) on a HiSeq2000 machine. 565,822,128 101b mate-pair reads (3 kb insert size, 24× genome coverage) were generated by Beckman Coulter Genomics (USA) on a HiSeq2000 machine. 707,375,498,101 bases paired-end reads (200 bp and 500 bp insert size, 22× and 4× genome coverage, respectively) and 31,547,468,250 bp MiSeq reads (400 bp insert size, 3× genome coverage) were generated by INRA EPGV (Evry, France) by using sequencing facilities of IG-CNG (CEA). Read quality was checked using FastQC (https://www.bioinformatics.babraham.ac.uk/projects/fastqc/).

### RNA sampling and sequencing

RNAs from F2 plants were extracted from 12 different tissues. Tissues and culture conditions are described in Additional file 1: Table S3. For each tissue, RNA was isolated from two independent pools (biological replicates), each combining material from at least 2 different plants. RNA was isolated with TRIzol® Reagent (Life Technologies) followed by a DNAse treatment and column purification (Qiagen RNAeasy) using manufacturers’ recommendations. RNA quantity was measured by Nanodrop analysis (Thermo Scientific) and RNA quality was assessed with an Agilent 2100 bioanalyzer. The cDNA of the biological replicate 1 was sequenced by the company GATC, the cDNA of the biological replicate 2 by the company COGENICS. Library construction (conversion of RNA into cDNA) involved oligoT columns and random priming (GATC) or an rRNA depletion kit and random priming (COGENICS). All samples were sequenced with single-end reads except four samples for which additional paired-end reads were generated (insert size 200 b), see Additional file 1: Table S3 for details.

### F2 whole genome assembly

The whole set of F2 reads was used for whole genome assembly (WGA). Read pre-processing was performed before assembly; N-containing reads were removed (Prinseq v0.20.3 [56], as well as adaptor-containing reads (Cutadapt v1.2.1 [57]). Putative human, fungal and bacterial contamination reads were discarded (Deconseq v0.4.2 [58]). Read pairs were synchronized using an in-house script. Read error correction was applied on cleaned data using SOAPec v2.01 (http://soap.genomics.org.cn/). Cleaned overlapping MiSeq reads were merged using FLASH v1.2.6 [59].

De novo assembly and scaffolding was performed with ABySS v1.3.5 (settings *n* = 3, k = 81) [60]. Scaffolding was further improved using the SSPACE scaffolder [61]. Gaps on the final assemblies were filled using 2 runs of GapFiller v1–11 (m = 90, o = 3, d = 1000) [62]. The WGA quality was evaluated in two ways. First, F2 WGA was aligned onto three full F2 BACs sequences (C. Vitte personal communication) with the NUCmer script [63] to evaluate contig contiguity and contigs order and orientation in scaffolds. Alignments were graphically represented using 1) mummerplot and 2) GEvo (https://genomevolution.org/coge/GEvo.pl). Second, a subset of F2 Illumina paired-end reads (40X) was aligned to F2 WGA using Stampy and a 24X depth F2 Illumina mate-pair reads (3 kb) was aligned to F2 WGA using SMALT 0.7.4 (http://www.sanger.ac.uk/science/tools/smalt-0), which aligns independently each read of a pair. Completeness of genic space was evaluated by searching a set of reference genes using CEGMA v2.5 [36] and BUSCO v1.22 [37] .

### Detection of genome sequences not shared between B73 and F2 using read mapping

The bioinformatics workflow for B73/F2 pangenome building is summarized in Additional file 8: Figure S7. Structural variants showing presence in F2 and absence in B73 were detected using Pindel v0.2.4 [64] and BreakDancer v1.4.5 [65]. First, B73 reads (40X dataset downloaded from NCBI Sequence Read Archive (SRA: SRR404240)) were aligned using Stampy onto F2 WGA. Aligned reads were then used as input for Pindel and BreakDancer, with a requirement of minimum local coverage of 3 reads with Q20 mapping quality. To discard possible false positives SVs due to F2 assembly errors or presence of stretches of Ns, we then performed the same analysis using reads from F2 with no SV as expectation (40× from our original dataset), and variants supported by both B73 and F2 reads were discarded. Variants smaller than 1 kb were not used for further analysis. Some F2 regions deleted in B73 may nevertheless be covered by B73 reads from other very similar copy in B73 and should not be classified as F2-specific. Therefore, only B73 deletions with B73 reads coverage <5X over 70% of SV size were considered as F2-specific and other deletions were filtered out. A fraction of F2 WGA scaffolds was poorly covered by B73 reads (total coverage < 20% of scaffold size) and could not be subjected to SV detection. When these scaffolds passed the F2-specificity criteria (B73 reads coverage <5X over 70% of scaffold size) they were classified as “incomplete PAVs”. All F2-specific sequences identified where then filtered to exclude those with potential assembly error. Only F2-specific sequences with F2 properly-mapped paired-end reads (correct read orientation and correct insert size) covering > 90% of PAV size were retained for this study. F2 properly-mapped paired-end reads rather than 2 kb F2 mate-pair reads were used as many PAVs are not long enough to exhibit a good coverage with mate-pair reads. The same procedure was used to identify regions present in B73 genome but absent from F2 genome. Note that no incomplete B73-specific region could be evidenced as the B73 genome is made of pseudomolecules and not unassembled scaffolds. Anchoring of F2-specific sequences to the B73 reference sequence was performed using a combination of YAHA v0.1.82 [66] and AGE v0.4 [67]. For anchored PAVs, microhomology stretches were extracted from AGE outputs.

### Construction of a B73/F2 pan-genome sequence

A B73/F2 pan-genome was created by adding the novel F2 sequences to the public B73 genome sequence v2 (http://www.genome.arizona.edu/modules/publisher/item.php?itemid=16).

F2-specific sequences were concatenated and separated by stretches of 100Ns. F2-specific sequences anchored and unanchored to the B73 genome were concatenated separately, leading to 2 distinct fasta sequences referred to as “anchored” and “unanchored”, respectively (Additional file 8: Figure S7).

### Sequence annotation of novel F2 regions and expression of novel F2 genes

Novel F2-specific sequences were annotated for genes and transposable elements. F2-specific regions were searched for TE using RepeatMasker (A.F.A. Smit, R. Hubley & P. Green RepeatMasker at http://repeatmasker.org) with database Repbase RELEASE 20150807 [68]. Gene annotation was based on mRNA-seq data analysis. mRNA-seq from B73, MO17, EP1, KUI3 and F331 leaves (C. Vitte, personal communication) and from each of the 12 F2 tissue samples from this study were subjected to sequence quality checking with Fastqc (https://www.bioinformatics.babraham.ac.uk/projects/fastqc/). Contaminant sequences were removed from the raw data using cutadapt (link: https://cutadapt.readthedocs.io/en/stable/, version: v1.0) with options: --discard –O 10 –q 1 –m 24). Cutadapt was also used to trim adapter sequences from the remaining reads (options: -O 10 –q 10 –n 2 –m 24). Reads pairs were synchronized using trim_galore.pl (link: http://www.bioinformatics.babraham.ac.uk/projects/trim_galore/, version: v0.2.7), with parameters –stringency 50 –q 10 –length 24 –phred 33. Overall, throughout this cleaning process, 10% of bases were removed from the initial raw data. mRNA-seq reads were mapped to the B73/F2 pan-genome sequence using TopHat v2.0.13 [69] with default settings. Genes prediction was based on transcriptome assembly with Cufflinks v2.2.1 [70] with default settings for each library. Transcriptomes were merged with the maize Filtered Gene Set (FGS) using Cuffmerge to build a B73/F2 pan-transcritome. FPKMs were computed using CuffNorm [70]. Genes overlapping RepeatMasker-based TE annotations over > 80% of their length were discarded. Novel F2 genes with CDS coding proteins with over 100 aa, with no significant blast hit (> 95% identity over 90% of the query length) with B73 sequences (NCBI no-redundant nucleotide bank) and FPKM > 1 at least in one library were selected for further expression analysis. F2 novel CDS were searched for known protein domains and sequence similarity using InterProscan 5 [71] and Blastp with the UniProt protein sequence database.

### Detection of SNPs in the B73/F2 pan-genome

One hundred one bp Illumina paired-end reads from a set of 23 maize inbred lines were aligned to the B73/F2 pan-genome to discover SNPs in B73/F2 shared regions as well as in F2-specific regions. This set of lines was provided by the Cornfed program (A. Charcosset, personal communication) and had been chosen to maximize the genetic diversity of American and European temperate maize (Additional file 1: Table S7) [54]. B73 and F2 reads used previously (sampled to 20X) were also included as controls. Reads were aligned against the B73/F2 pan-genome sequence using Stampy v1.0.21 [72] and PCR duplicates were removed using SAMtools rmdup v0.1.18 [73]. SNPs were detected using SAMtools mpileup (-B) and VarScan v 2.3.6 (−-min-coverage 3 --min-avg-qual 30 --min-var-freq 0.9 --*p*-value 0.05) [74]. SNPs corresponding to Ns were discarded. For B73/F2 shared regions, SNPs with a different call between the B73 reference sequence and B73 Illumina reads were also discarded. For F2 novel regions, SNPs showing a different call in F2 reference and F2 Illumina reads were discarded, as well as SNPs covered by B73 reads. Our SNP discovery pipeline was tested using genotyping from a 50 k Illumina Infinium array [75], and showed a high agreement rate (> 99%).

### Genotyping presence/absence of F2 novel regions in a core set of 23 maize lines

Presence/absence of F2 novel regions in the Cornfed set of 23 maize lines was assessed using a clustering analysis based on reads count in PAV sequence. Each line was analyzed as follows. For each region i, we predicted whether i was present (i.e. belongs to cluster 1) or absent (belongs to cluster 0). Given Zi the cluster membership of region i to be determined, xi the copy number of region i measured through local sequencing depth of F2 and Yi the copy number of region i measured through local sequencing depth of the considered line, the relationship between xi and Yi is assumed to be linear (after square root transformation of the sequencing count data): Yi = a0 + b0xi + Ei if Zi = 0; Yi = a1 + b1xi + Ei if Zi = 1 (mixture model of regression), where a0 and b0 (resp. a1 and b1) are the intercept and slope of the linear relationship for regions of cluster 0 (resp. 1), and Ei is the error term. Errors are assumed to follow a Gaussian distribution with mean 0 and variance σ^2^, and to be independent. The goal of the analysis was then to estimate the coefficients (ak; bk; σ^2^k, k = 0, 1), and more importantly the posterior probabilities τik of each region i to belong to cluster k., Maximum Likelihood estimation was performed using the EM algorithm [76]. Regions were then classified as present or absent based on their estimated posterior probabilities τik. Misclassification error rate was accounted for by applying a Bayesian False Discovery Rate (BFDR) control procedure [77] with a nominal BFDR fixed at 0.01 (Additional file 9: Figure S8).

### Genetic diversity analysis

SNPs and PAVs scoring for the 23 maize lines (excluding missing data) were analyzed by Principal Component Analysis using the FactoMineR R package [78]. PAV frequencies were computed for each genetic group (European Flint, Northern Flint, Corn Belt Dent and Synthetic Stiff Stalk) and subjected to Hierarchical Clustering. Linkage Disequilibrium was computed using the PLINK software v1.90b3s [79], where LD decay is estimated by the squared correlations of allele frequency (*r*^2^) against distance between polymorphic sites. Average LD versus physical distance was plotted using the R software. Only anchored PAVs were used. Two types of analyses were made: (i) LD was computed between each PAV and SNPs from flanking regions. In this analysis, PAV polymorphisms were either coded as 0/1 or represented by internal SNPs. (ii) LD was computed individually within PAVs and in flanking regions from SNPs extracted from these regions. For LD computation in gene space and inter-genic space, two sets of 30 kb genomic regions either overlapping gene or not overlapping gene were sampled randomly and LD decay was computed between the most central SNP of each region and all other SNPs of the region. For each PAV, 20 kb upstream and downstream flanking regions were extracted and LD was computed between all SNPs.

## Additional files

Additional file 1: Supplementary tables. **Table S1.** FV2 genome size estimation by flow-cytometry analysis. **Table S2.** Validation of whole-genome de novo assembly using CEGMA and BUSCO. **Table S3.** RNA sequencing from this study. **Table S4.** Functional annotation of FV2 novel genes. **Table S5.** Genes present in B73 and deleted or partly-deleted in FV2. **Table S6.** Blast best hit for F2-specific genes against the NCBI NR sequences. **Table S7.** DNAseq used for SNP detection. (XLSX 89 kb)

Additional file 2: Figure S1. Quality analysis of F2 WGA in a gene-rich region. A. A MUMmer alignment showing that more than 44% of a 112.2 kb F2 BAC sequence from the *Bronze* locus is covered by 3 scaffolds from the F2 WGA. B. Nearly all genes of this region are assembled into one single scaffold while the two other scaffolds cover mostly non-genic DNA and TE. Gaps indicated by a star (A) or an orange box (B) delineate contigs of each scaffold. All contigs are correctly ordered and oriented even in non-genic regions. (PDF 464 kb)

Additional file 3: Figure S2. Quality analysis of F2 WGA in a transposon-rich region. A. A MUMmer alignment showing that a unique scaffold from the F2 WGA covers 62% of a 74.7 kb F2 BAC sequence from a gene-free region. B. While a 20 kb region is absent from the assembly, contigs delineated by gaps (indicated by a star (A) or an orange box (B)) are correctly ordered and oriented showing that the F2 WGA is able to correctly covers large regions encompassing mainly TEs. (PDF 245 kb)

Additional file 4: Figure S3. Quality analysis of F2 WGA in a transposon-rich region. A. A MUMmer alignment showing that more than 80% of a 116.7 kb F2 BAC sequence is covered by 5 scaffolds from the F2 WGA. B. With no exception, contigs delineated by gaps (indicated by a star (A) or an orange box (B)) are correctly ordered and oriented showing that the F2 WGA is able to correctly covers large regions encompassing TEs. (PDF 615 kb)

Additional file 5: Figure S4. Long-jump read coverage of F2 WGA and F2-specific sequences. F2 WGA was evaluated by mapping reads from a F2 3 kb mate-pair Illumina library (24X) on F2 scaffolds. Both reads of pairs were mapped independently with SMALT 0.7.4. A. Insert size distribution after mapping. Only pairs exhibiting an insert size ranging from 1.6 to 3 kb were considered as correctly mapped and used for subsequent evaluation. B. Scaffold coverage versus scaffold size. Coverage computation is based on properly mapped long jump reads with insert size ranging from 1.6 kb to 3 kb. As expected, small scaffolds are less efficiently covered by long jump reads. C. F2-specific scaffolds fraction shows an enrichment in well-covered scaffolds (> 70% coverage) compared to F2 WGA (B). (PDF 8588 kb)

Additional file 6: Figure S5. Size distribution of B73 and F2-specific sequences. All PAVs exhibit similar size distribution except F2 unanchored PAVs, which are more numerous and include larger sequences. (PDF 28 kb)

Additional file 7: Figure S6. Bioinformatics workflow of pan-genome sequence building. All steps are described in details in the method section. (PDF 702 kb)

Additional file 8: Figure S7. Example of PAVs genotyping using resequencing data. Classification of PAVs in inbred line EP1 based on information from 20X sequencing depth. Each dot represents one PAV. Green and red dots correspond to PAVs that are confidently classified as present and absent, respectively (80% of all PAVs). Black dots correspond to unclassified PAVs (20% of all PAVs). (PDF 279 kb)

Additional file 9: Classification of PAVs with mapped short-reads count (see Methods). (PDF 224 kb)

## Abbreviations

BDFR: Bayesian False Discovery Rate
CDS: Coding Domain Sequence
CEGMA: Core Eukaryotic Genes Mapping Approach
CGH: Comparative Genomic Hybridization
CNV: Copy Number Variant
DSBR: Double-Strand Break Repair
EM algorithm: Expectation Maximisation algorithm
EST: Expressed Sequence Tag
FPKM: Fragments Per Kilobase of transcript per Million fragments mapped
GBS: Genotyping by sequencing
GWAS: Genome-Wide Association Studies
HMM: Hidden Markov Model
LD: Linkage Disequilibrium
MMEJ: Microhomology-Mediated End Joining
NHEJ: Non-Homology End Joining
PAV: Presence-Absence Variant
SNP: Single Nucleotide Polymorphism
SV: Structural Variation
TE: Transposable Element
