# Supplementary figures and images for: Sequence analysis of European maize inbred line F2 provides new insights into molecular and chromosomal characteristics of presence/absence variants
Source: BMC Genomics. 2018 Feb 5;19:119. doi: 10.1186/s12864-018-4490-7 (PMC5800051; doi:10.1186/s12864-018-4490-7)

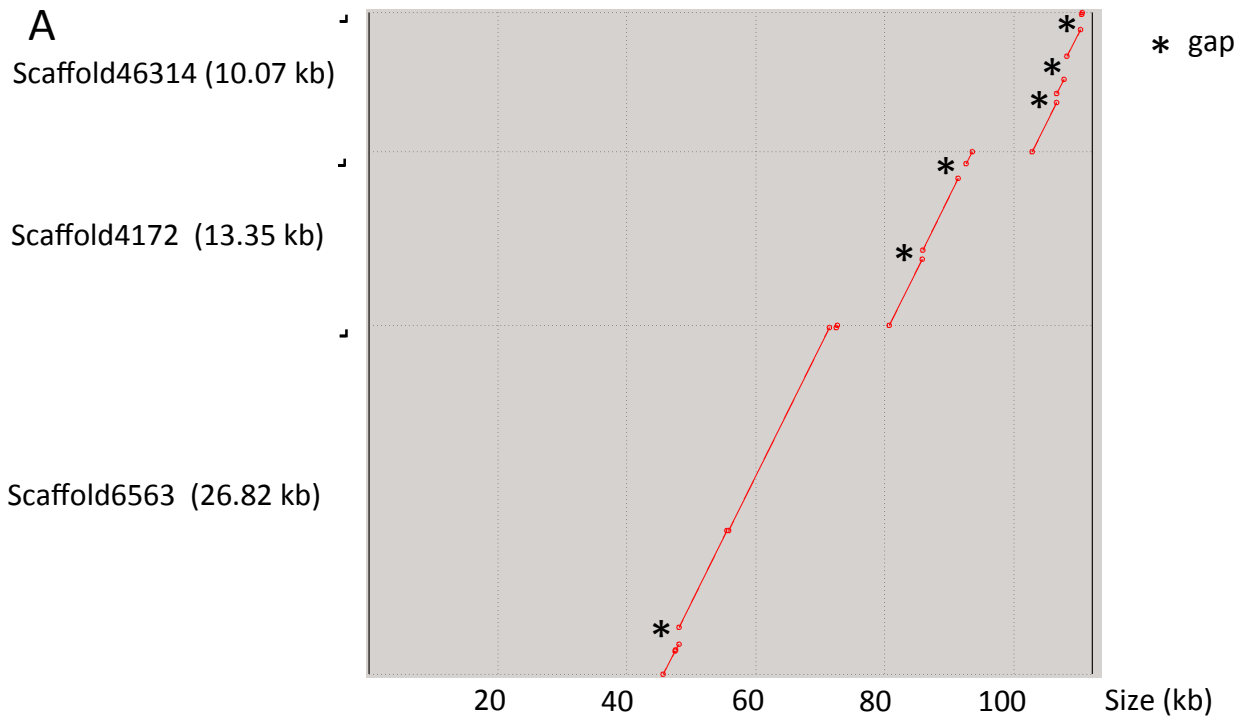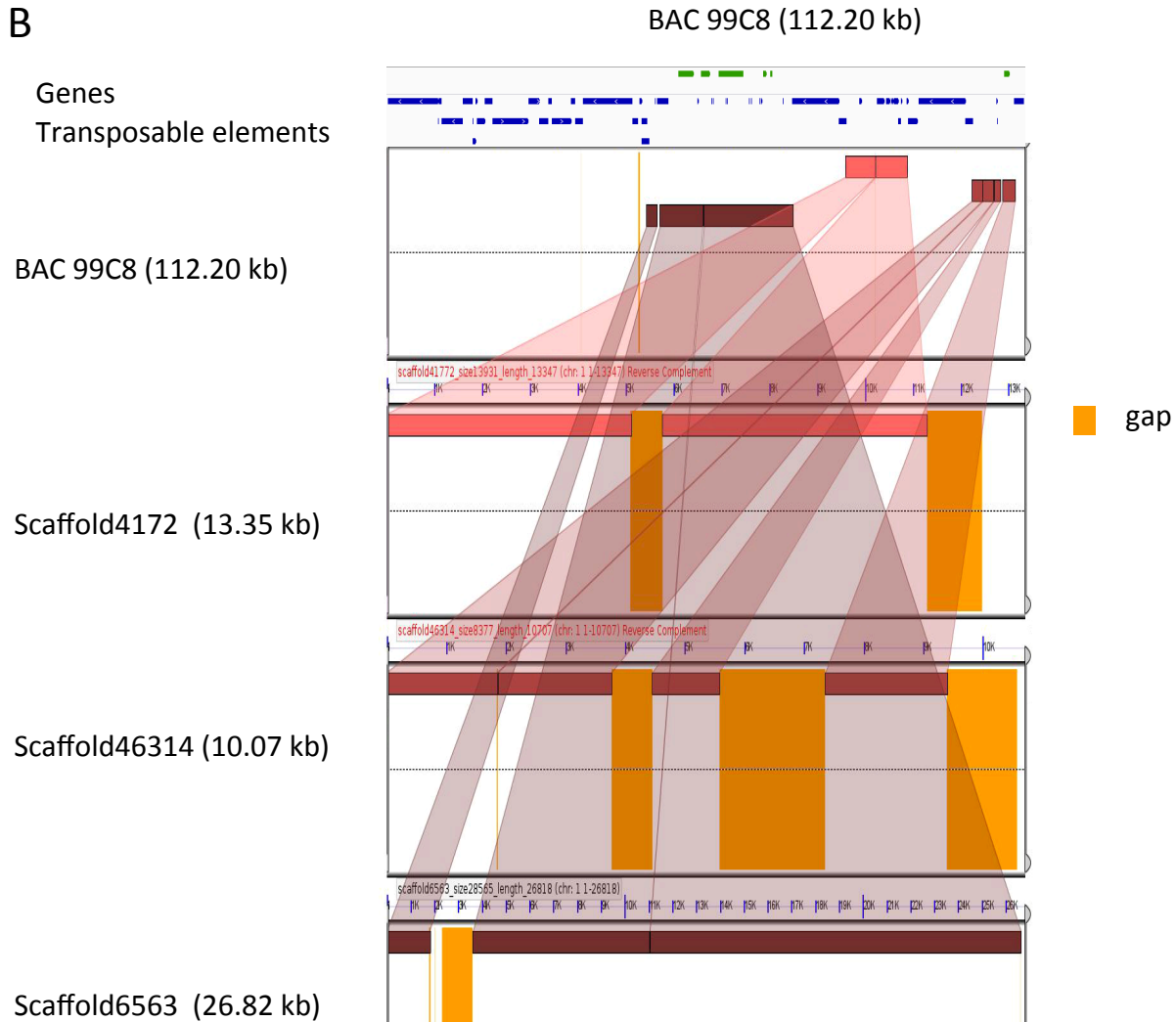

**Figure S1**

Supplement: Supplementary file 2 — Quality analysis of F2 WGA in a gene-rich region. A. A MUMmer alignment showing that more than 44% of a 112.2 kb F2 BAC sequence from the Bronze locus is covered by 3 scaffolds from the F2 WGA. B. Nearly all genes of this region are assembled into one single scaffold while the two other scaffolds cover mostly non-genic DNA and TE. Gaps indicated by a star (A) or an orange box (B) delineate contigs of each scaffold. All contigs are correctly ordered and oriented even in non-genic regions. (PDF 464 kb) [file 12864_2018_4490_MOESM2_ESM.pdf]

A

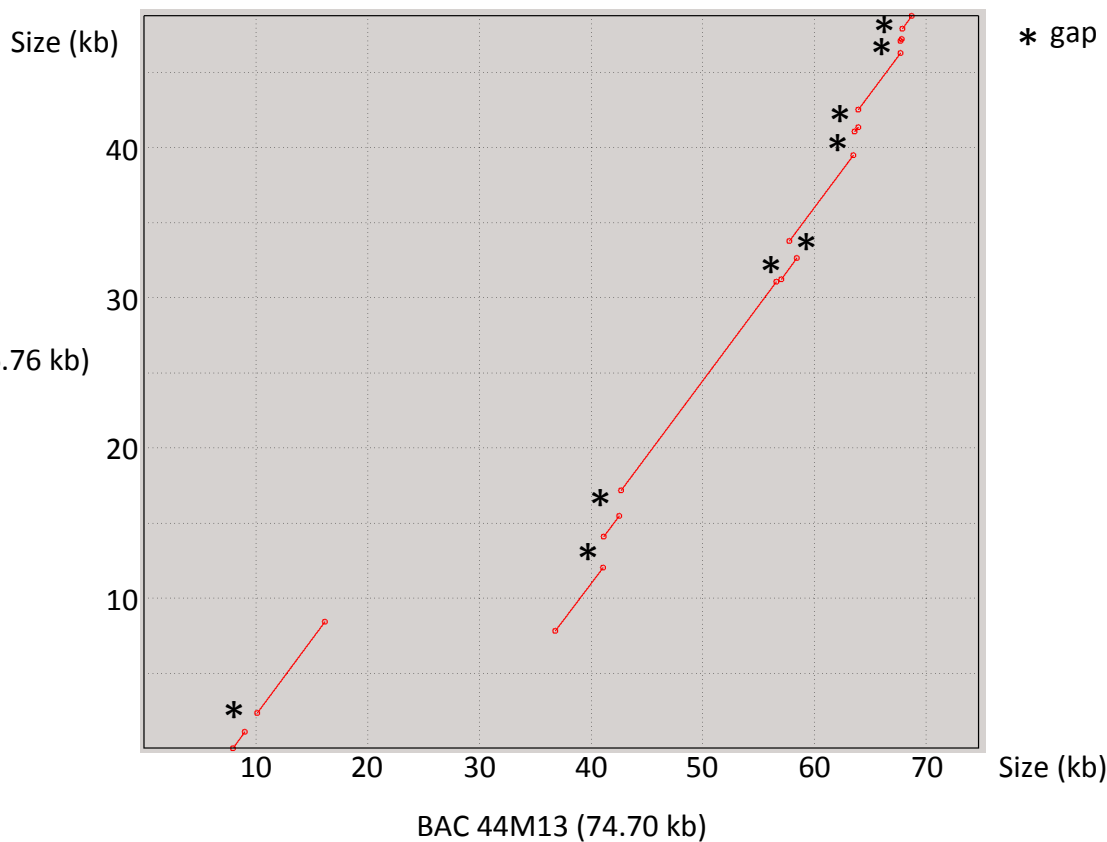

B

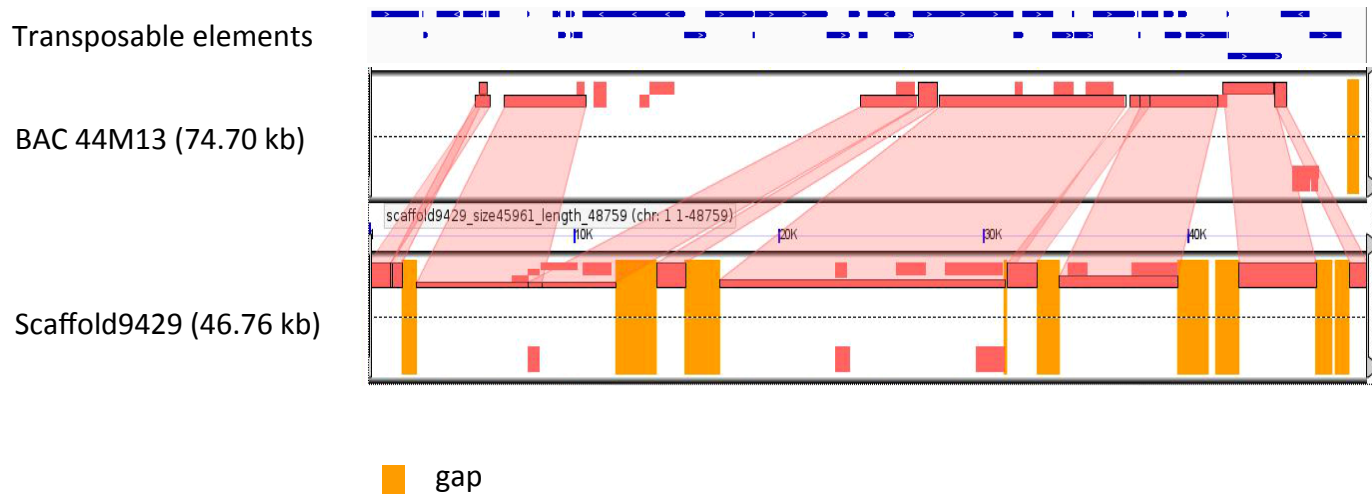

Figure S2

Supplement: Supplementary file 3 — Quality analysis of F2 WGA in a transposon-rich region. A. A MUMmer alignment showing that a unique scaffold from the F2 WGA covers 62% of a 74.7 kb F2 BAC sequence from a gene-free region. B. While a 20 kb region is absent from the assembly, contigs delineated by gaps (indicated by a star (A) or an orange box (B)) are correctly ordered and oriented showing that the F2 WGA is able to correctly covers large regions encompassing mainly TEs. (PDF 245 kb) [file 12864_2018_4490_MOESM3_ESM.pdf]

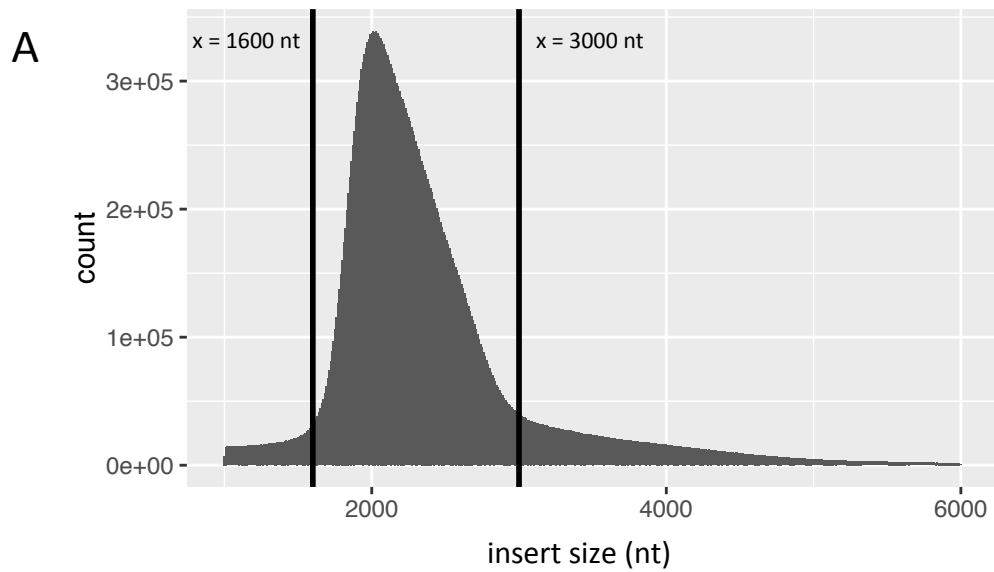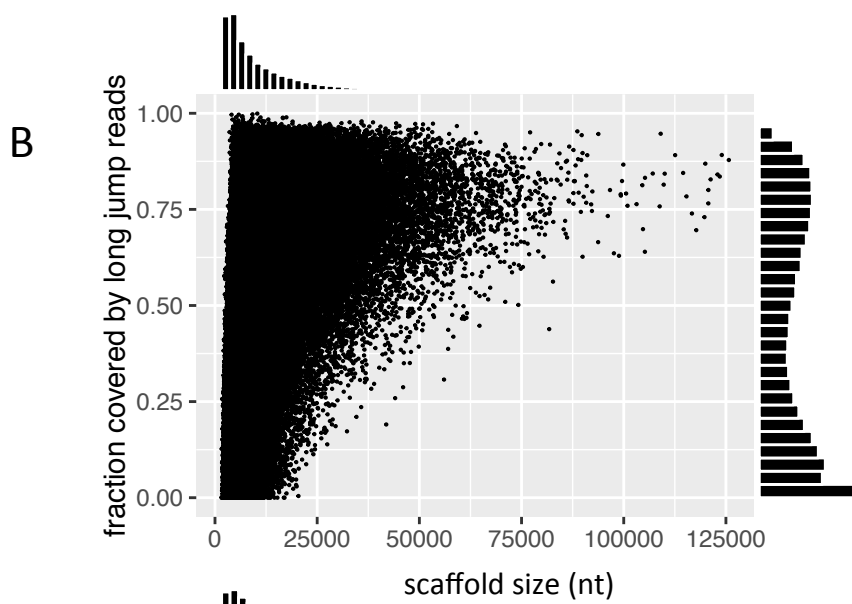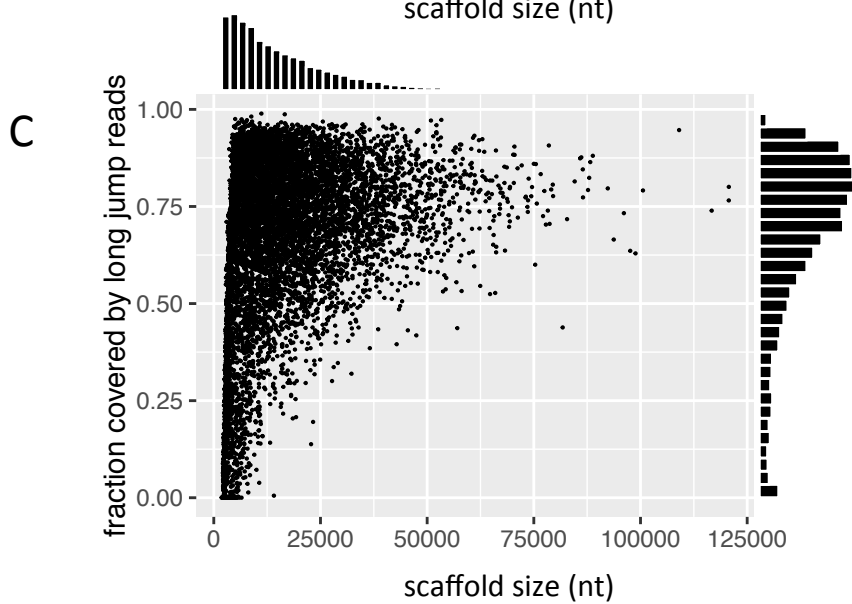

**Figure S4**

Supplement: Supplementary file 5 — Long-jump read coverage of F2 WGA and F2-specific sequences. F2 WGA was evaluated by mapping reads from a F2 3 kb mate-pair Illumina library (24X) on F2 scaffolds. Both reads of pairs were mapped independently with SMALT 0.7.4. A. Insert size distribution after mapping. Only pairs exhibiting an insert size ranging from 1.6 to 3 kb were considered as correctly mapped and used for subsequent evaluation. B. Scaffold coverage versus scaffold size. Coverage computation is based on properly mapped long jump reads with insert size ranging from 1.6 kb to 3 kb. As expected, small scaffolds are less efficiently covered by long jump reads. C. F2-specific scaffolds fraction shows an enrichment in well-covered scaffolds (> 70% coverage) compared to F2 WGA (B). (PDF 8588 kb) [file 12864_2018_4490_MOESM5_ESM.pdf]

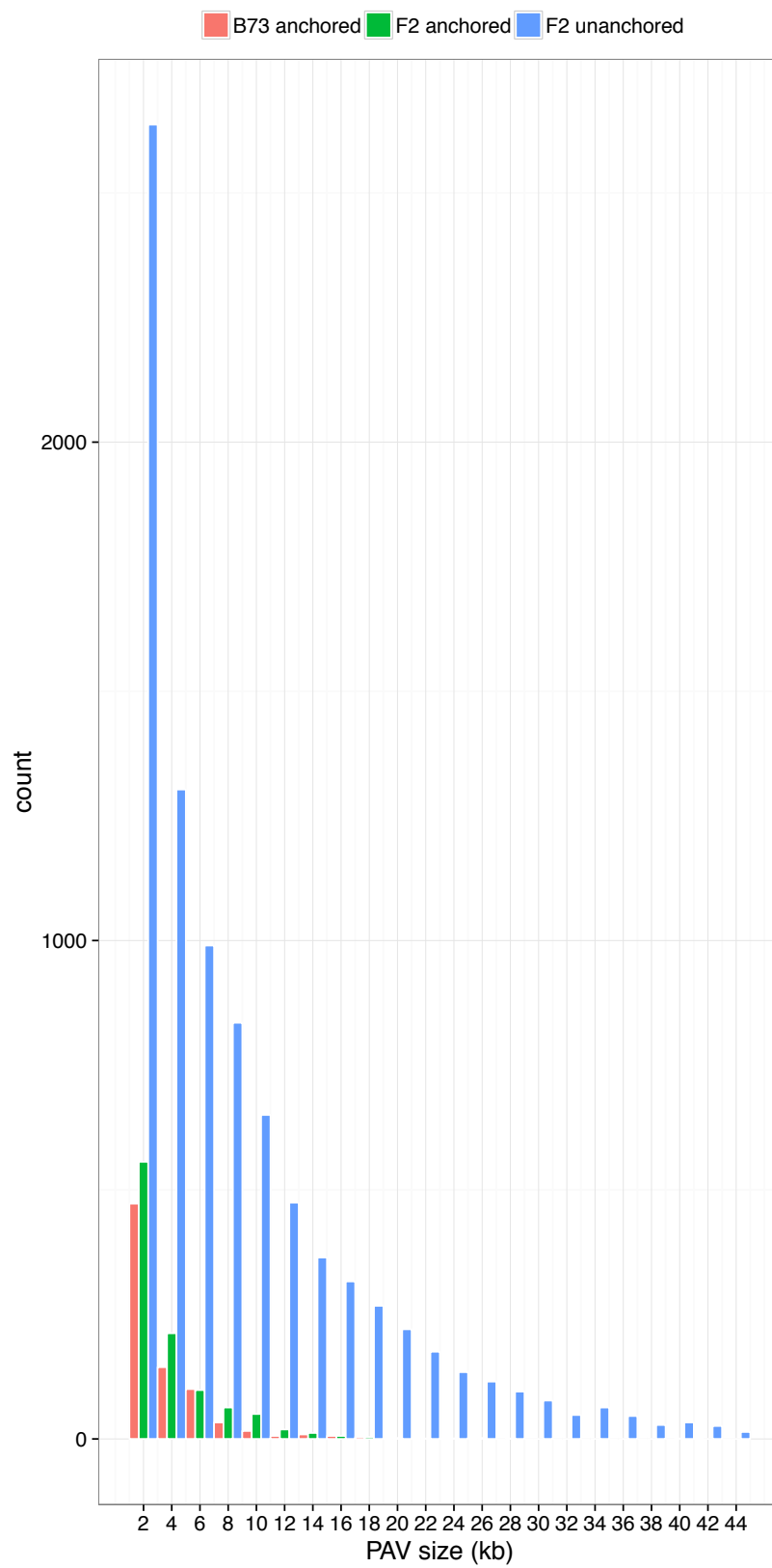

Figure S5

Supplement: Supplementary file 6 — Size distribution of B73 and F2-specific sequences. All PAVs exhibit similar size distribution except F2 unanchored PAVs, which are more numerous and include larger sequences. (PDF 28 kb) [file 12864_2018_4490_MOESM6_ESM.pdf]

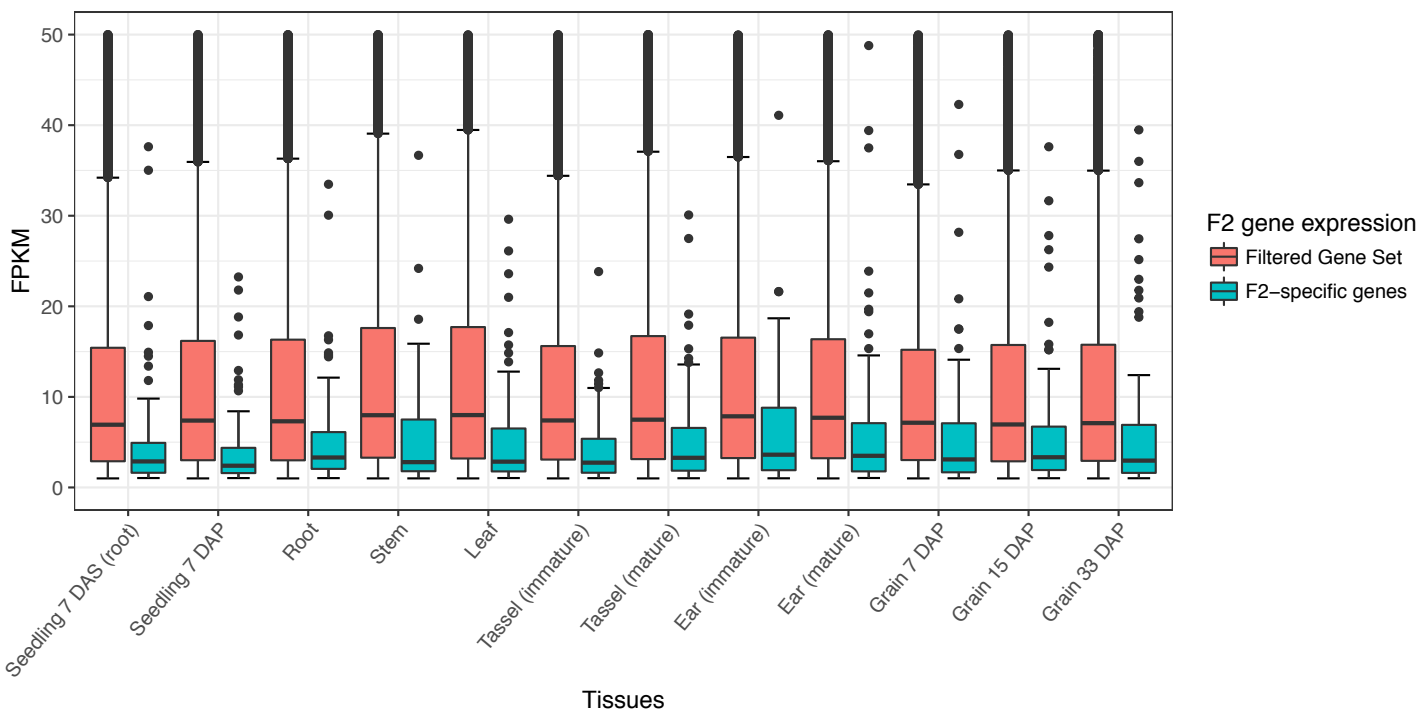

**Figure S6**

Supplement: Supplementary file 7 — Bioinformatics workflow of pan-genome sequence building. All steps are described in details in the method section. (PDF 702 kb) [file 12864_2018_4490_MOESM7_ESM.pdf]

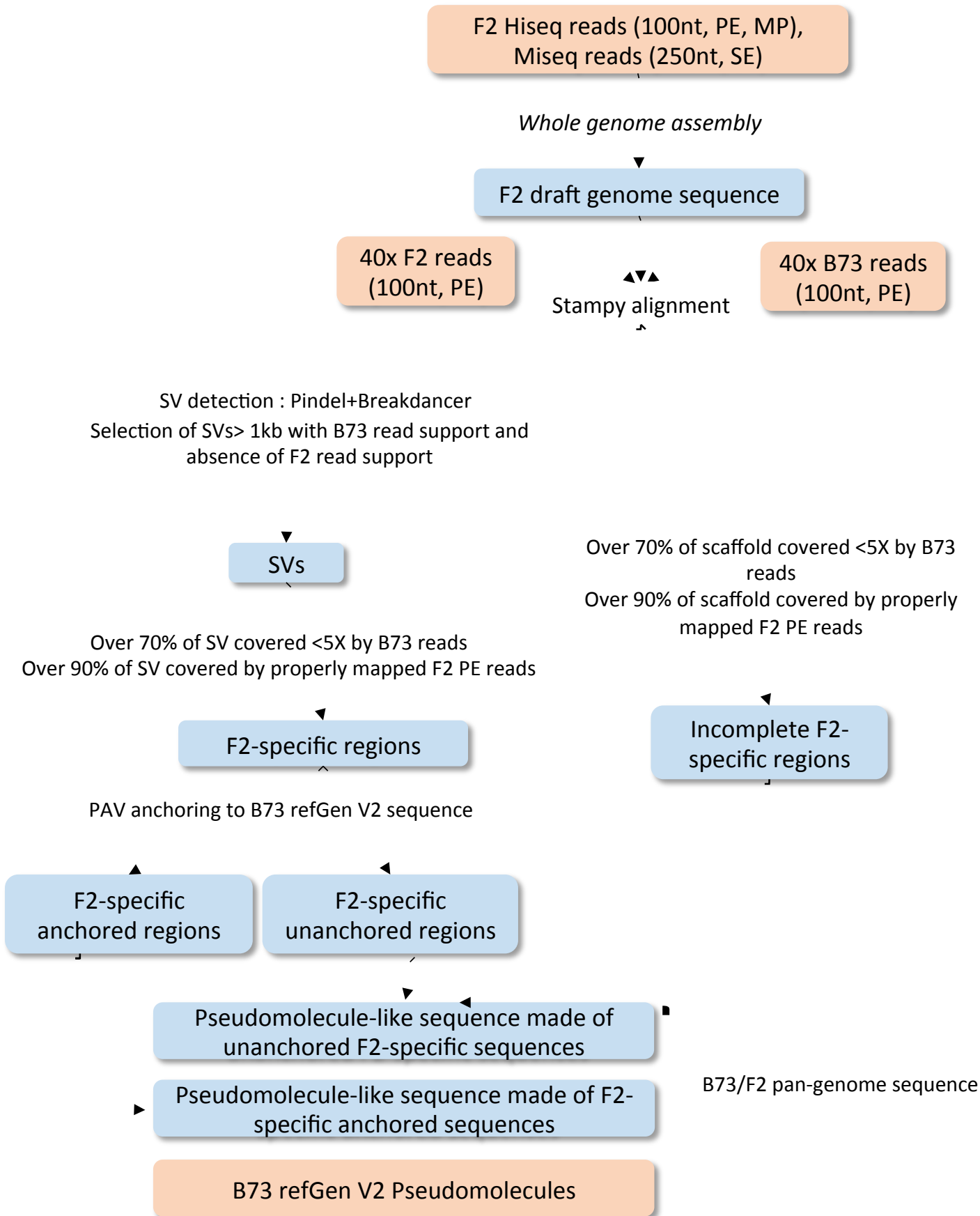

**Figure S7**

Supplement: Supplementary file 8 — Example of PAVs genotyping using resequencing data. Classification of PAVs in inbred line EP1 based on information from 20X sequencing depth. Each dot represents one PAV. Green and red dots correspond to PAVs that are confidently classified as present and absent, respectively (80% of all PAVs). Black dots correspond to unclassified PAVs (20% of all PAVs). (PDF 279 kb) [file 12864_2018_4490_MOESM8_ESM.pdf]

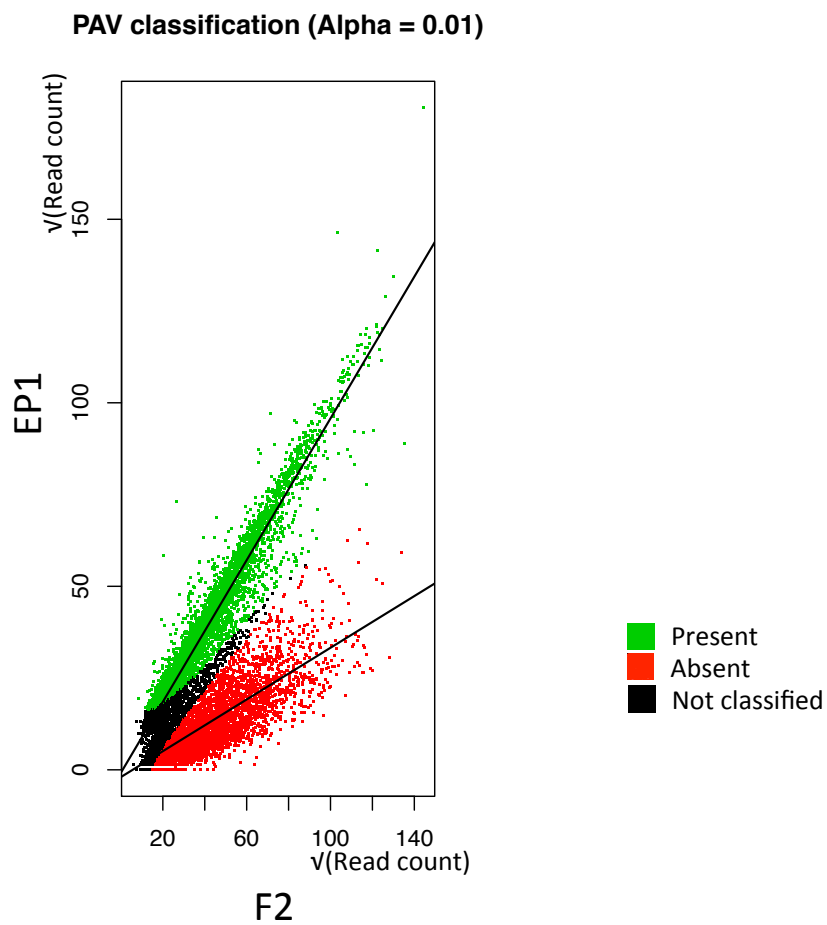

**Figure S8**

Supplement: Supplementary file 9 — Classification of PAVs with mapped short-reads count (see Methods). (PDF 224 kb) [file 12864_2018_4490_MOESM9_ESM.pdf]
