# Supplementary material for: Sequence analysis of European maize inbred line F2 provides new insights into molecular and chromosomal characteristics of presence/absence variants
Source: BMC Genomics. 2018 Feb 5;19:119. doi: 10.1186/s12864-018-4490-7 (PMC5800051; doi:10.1186/s12864-018-4490-7)
Supplement: Supplementary file 4 — Quality analysis of F2 WGA in a transposon-rich region. A. A MUMmer alignment showing that more than 80% of a 116.7 kb F2 BAC sequence is covered by 5 scaffolds from the F2 WGA. B. With no exception, contigs delineated by gaps (indicated by a star (A) or an orange box (B)) are correctly ordered and oriented showing that the F2 WGA is able to correctly covers large regions encompassing TEs. (PDF 615 kb) [file 12864_2018_4490_MOESM4_ESM.pdf]

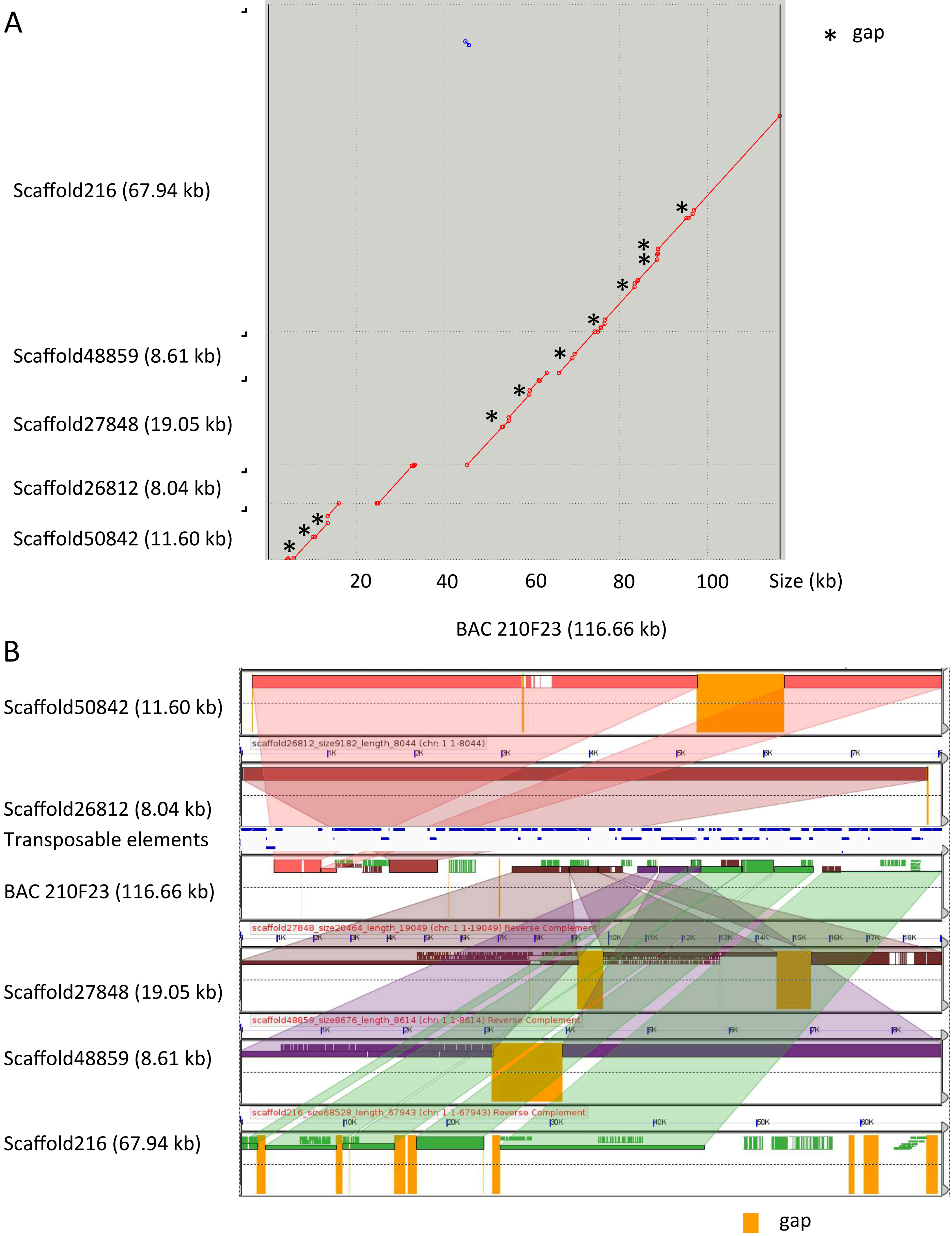
A
gap
Scaffold216 (67.94 kb)
Scaffold48859 (8.61 kb)
Scaffold27848 (19.05 kb)
Scaffold26812 (8.04 kb)
Scaffold50842 (11.60 kb)
20
40
60
80
100
Size (kb)
BAC 210F23 (116.66 kb)
B
Scaffold50842 (11.60 kb)
scaffold26812_size9182_length_8044 (chr: 1 1-8044)
Scaffold26812 (8.04 kb)
Transposable elements
BAC 210F23 (116.66 kb)
scaffold27848_size20464_length_19049 (chr: 1 1-19049) Reverse Complement
Scaffold27848 (19.05 kb)
scaffold48859_size8676_length_8614 (chr: 1 1-8614) Reverse Complement
Scaffold48859 (8.61 kb)
scaffold216_size68528_length_67943 (chr: 1 1-67943) Reverse Complement
Scaffold216 (67.94 kb)
gap

Figure S3
